# Supplementary material for: Design, Synthesis and Anticancer Profile of New 4-(1H-benzo[d]imidazol-1-yl)pyrimidin-2-amine-Linked Sulfonamide Derivatives with V600EBRAF Inhibitory Effect
Source: Int J Mol Sci. 2021 Sep 28;22(19):10491. doi: 10.3390/ijms221910491 (PMC8508980; doi:10.3390/ijms221910491)
Supplement: Supplementary file 1 [file ijms-22-10491-s001.zip › ijms-1372881-supplementary.pdf]

Design, Synthesis and Anticancer Profile of New 4-(1*H*-benzo[*d*]imidazol-1-yl) pyrimidin -2-amine Linked Sulfonamide Derivatives with V600EBRAF Inhibitory Effect

Mohammed S. Abdel-Maksoud[1,†,*], Ahmed A. B. Mohamed[2,†], Rasha M. Hassan[1], Mohamed A. Abdelgawad[3], Garri Chilingaryan[4], Samy Selim[5], Mohamed S. Abdel-Bakky[6], Mohammad M. Al-Sanea[3,*]

1 Medicinal &Pharmaceutical Chemistry Department, Pharmaceutical and Drug Industries Research Division, National Research Centre (NRC), (ID: 60014618), P.O. , Dokki, Giza 12622, Egypt, ph_ss@hotmail.com, rashahassan_pharma@yahoo.ca

2 Department of Medicinal Chemistry, Faculty of Pharmacy, Mansoura University, Mansoura 35516, Egypt, ahmed_smt@yahoo.com

3 Department of Pharmaceutical Chemistry, College of Pharmacy, Jouf University, Sakaka, Aljouf 72341, Saudi Arabia, mhmdgwd@ju.edu.sa

4 Institute of Molecular Biology of NAS, Yerevan 0014, Armenia, g_chilingaryan@mb.sci.am

5 Department of Clinical Laboratory Sciences, College of Applied Medical Sciences, Jouf University, Sakaka, Aljouf 72341, Saudi Arabia, sabdulsalam@ju.edu.sa

6 Department of Pharmacology and Toxicology, College of Pharmacy, Qassim University, Qassim 51452 , Saudi Arabia, m.abdelbakky@qu.edu.sa

* Correspondence: mmalsanea@ju.edu.sa, ms.abdel-maksoud@nrc.sci.eg

† These authors are equally contributed to this work.

Table of content

Content	page
Title	1s
Table of content	2s
Figure 1s. Map heat analysis of target compounds **12a-g** over NCI 60 cell lines	3s
Figure 2s. Map heat analysis of target compounds **12h-n** over NCI 60 cell lines	4s
Table 1s : Percent growth over 60 cell lines for ethyl linker containing derivatives **12a-g**	5s-6s
Table 2s : Percent growth over 60 cell lines for propyl linker containing derivatives **12h-n**	7s-8s
NCI data	9s-22s
Spectral data	23s-49s
NCI standard procedure for 60 cell lines assay	50s

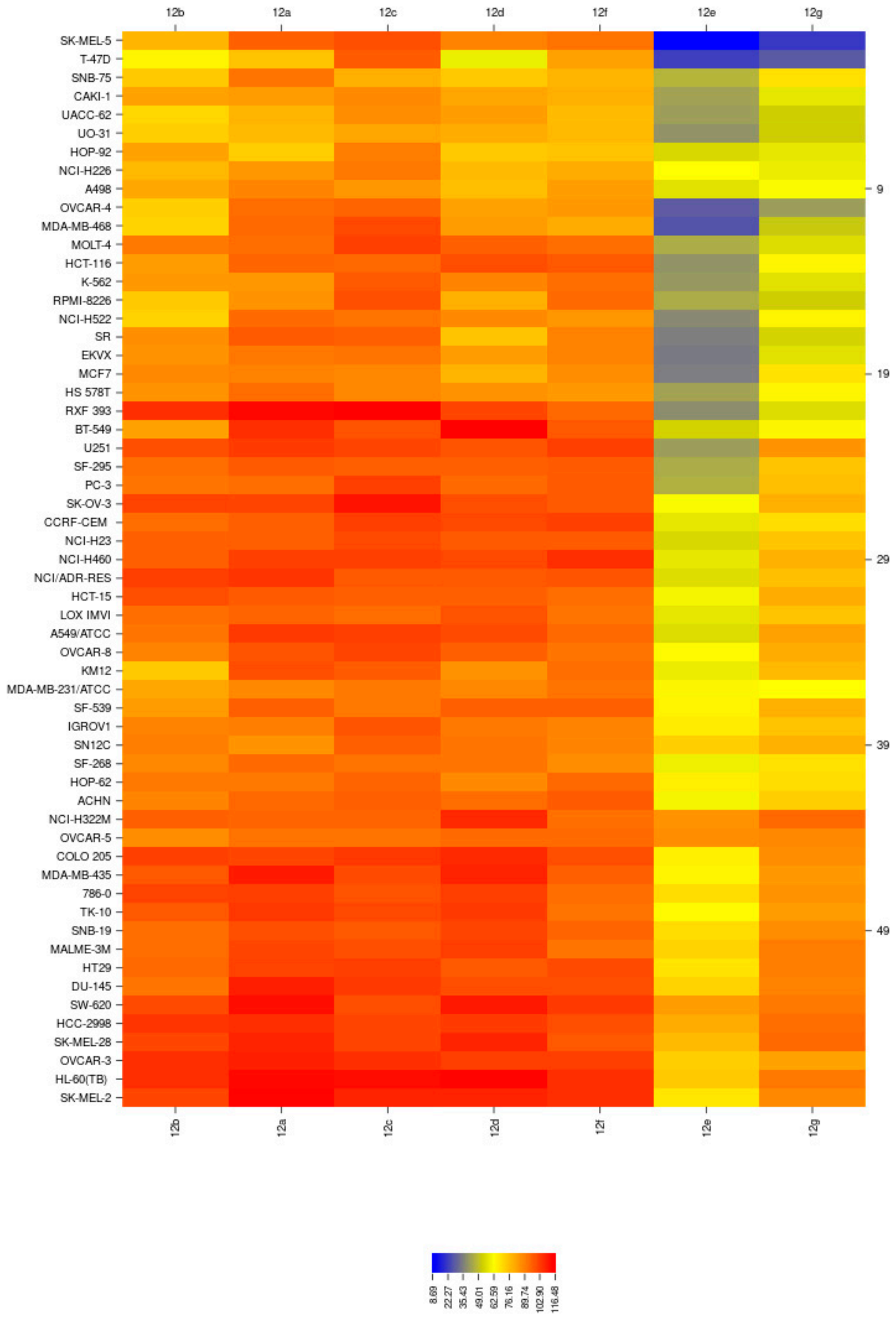

Figure 1s. Map heat analysis of target compounds **12a-g** over NCI 60 cell lines.

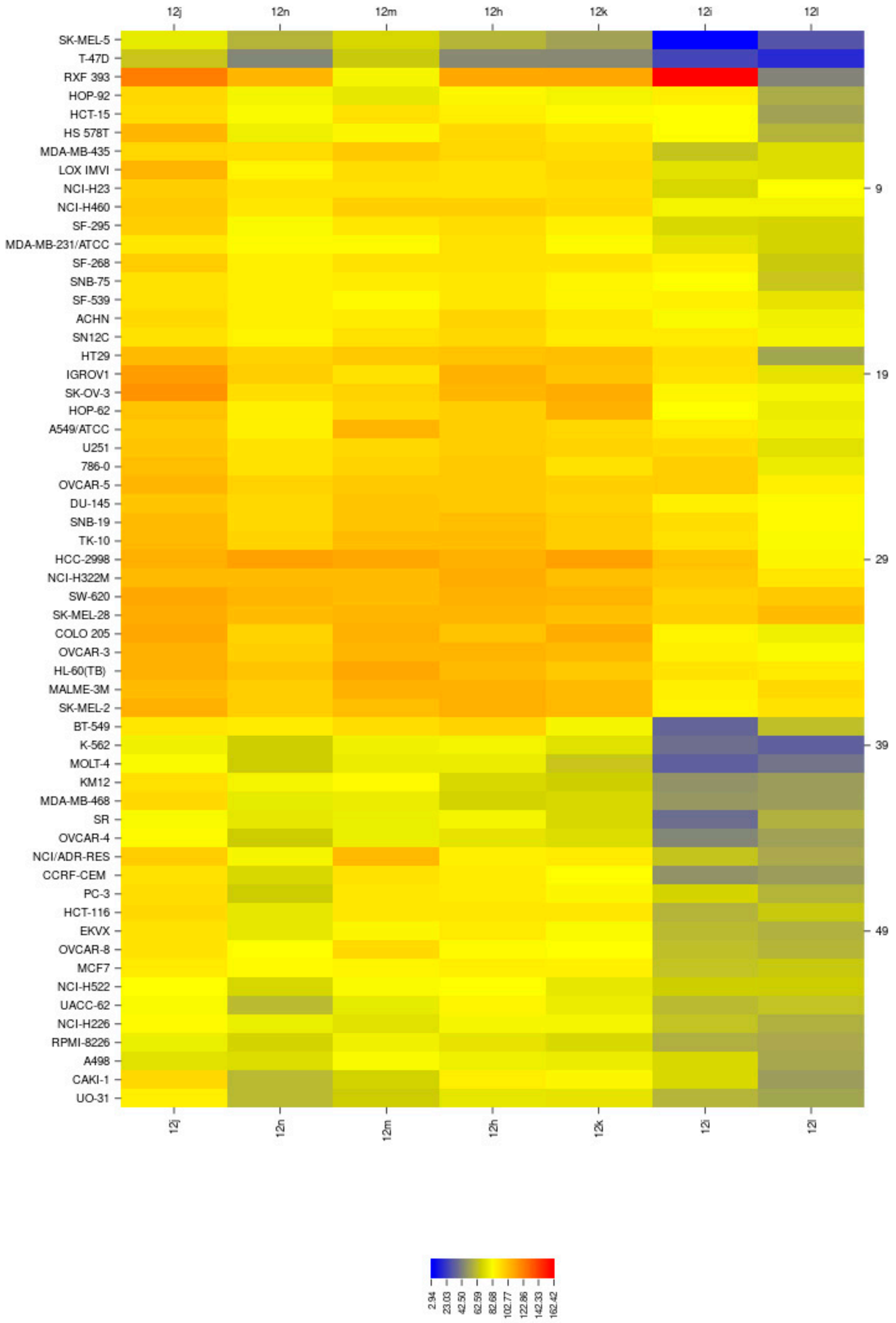

Figure 2s. Map heat analysis of target compounds **12h-n** over NCI 60 cell lines.

Table 1s : Percent growth over 60 cell lines for ethyl linker containing derivatives **12a-g**.

Subpanel	12a	12b	12c	12d	12e	12f	12g
**Leukemia**							
CCRF-CEM	96.04	93.45	102.97	100.92	57.64	102.48	69.49
HL-60(TB)	112.89	106.15	112.54	114.36	73.82	105.97	91.22
K-562	84.78	84.05	97.63	88.40	40.82	92.95	**56.14**
MOLT-4	93.72	90.46	102.61	95.86	45.17	93.29	**55.29**
RPMI-8226	84.93	73.62	99.40	79.75	44.94	94.04	52.51
SR	97.54	85.84	96.34	75.08	35.52	89.16	53.63
**Non-Small Cell Lung Cancer**							
A549/ATCC	103.72	91.26	102.78	100.15	55.43	94.62	82.36
EKVX	91.22	85.24	92.22	83.36	34.69	89.19	56.10
HOP-62	90.71	90.88	95.36	87.17	66.07	94.60	69.62
HOP-92	73.31	82.27	90.16	74.24	54.55	75.40	57.86
NCI-H226	84.57	77.55	91.12	76.53	62.71	80.60	58.69
NCI-H23	96.41	95.81	100.70	97.30	54.55	96.92	74.63
NCI-H322M	94.88	95.62	95.27	107.00	85.30	92.58	94.38
NCI-H460	102.66	96.29	102.89	100.58	57.80	106.11	79.00
NCI-H522	93.85	71.82	92.11	87.18	38.17	84.44	64.56
**Colon Cancer**							
COLO 205	101.30	102.41	104.07	107.01	64.92	99.76	86.83
HCC-2998	105.81	104.40	101.21	103.48	80.46	99.72	93.09
HCT-116	94.63	83.58	94.61	99.76	40.08	97.98	64.09
HCT-15	97.00	100.06	96.64	95.73	60.72	93.72	80.22
HT29	101.92	94.36	102.72	97.17	68.00	100.15	90.15
KM12	99.99	73.43	97.07	85.44	58.72	93.20	77.40
SW-620	112.27	100.16	100.05	110.11	83.49	103.20	90.84
**CNS-Cancer**							
SF-268	94.07	88.19	92.34	91.24	59.50	86.72	68.77
SF-295	97.28	93.57	96.65	96.48	44.68	96.98	75.49
SF-539	96.07	83.72	91.15	96.18	64.84	96.17	79.68
SNB-19	99.64	92.97	97.72	101.26	69.76	95.30	86.81
SNB-75	91.75	73.63	79.37	74.11	46.77	78.79	68.99
U251	103.34	99.55	101.61	98.60	42.41	102.85	85.15
**Melanoma**							
LOX IMVI	95.05	93.25	93.49	99.07	57.44	91.67	75.18
MALME-3M	101.04	93.70	99.54	102.82	71.37	91.72	90.24
M14	---	---					
MDA-MB-435	109.49	97.74	100.22	108.45	64.37	96.62	84.11
SK-MEL-2	114.98	101.47	108.54	107.85	67.76	105.98	87.74
SK-MEL-28	108.05	101.20	101.46	108.16	77.19	97.92	94.43

SK-MEL-5	95.82	78.02	99.44	89.14	8.48	92.21	20.80
UACC-62	78.31	71.28	86.37	82.77	42.41	76.59	53.03
**Ovarian Cancer**							
IGROV1	89.88	89.26	98.20	90.72	66.39	88.99	75.18
OVCAR-3	108.63	106.17	105.65	102.75	72.90	102.87	82.02
OVCAR-4	93.41	72.29	95.25	82.22	28.42	84.28	42.15
OVCAR-5	92.49	86.58	91.71	93.95	86.24	93.88	87.83
OVCAR-8	98.02	89.01	101.55	95.60	63.50	91.47	79.83
NCI/ADR-RES	104.47	102.88	97.81	97.63	55.91	98.74	75.74
SK-OV-3	101.19	102.17	111.56	99.94	62.23	97.45	79.46
**Renal Cancer**							
786-0	102.76	102.21	98.98	102.91	69.65	92.97	85.41
A498	88.60	80.70	84.78	76.27	56.61	82.86	62.06
ACHN	94.21	88.29	96.40	93.63	60.80	97.14	72.98
CAKI-1	83.72	82.01	87.40	81.55	43.10	79.60	57.53
RXF 393	113.30	105.90	116.69	100.99	38.25	94.57	55.39
SN12C	85.07	89.69	96.64	91.48	72.54	88.51	79.03
TK-10	104.20	97.82	100.85	103.12	63.40	92.50	83.69
UO-31	77.03	72.63	81.59	79.80	39.85	77.26	52.03
**Prostate Cancer**							
PC-3	93.53	91.34	103.00	93.82	45.46	97.53	76.11
DU-145	109.21	91.55	103.71	99.93	71.82	99.91	89.27
**Breast Cancer**							
MCF7	89.32	87.73	87.90	78.37	34.88	86.18	67.90
MDA-MB-231/ATCC	87.62	81.83	90.55	88.16	63.96	92.06	62.50
HS 578T	93.50	85.50	88.06	85.69	42.89	84.13	64.25
BT-549	105.93	82.22	99.29	115.11	53.06	97.83	63.69
T-47D	75.44	64.38	97.07	59.10	22.34	82.35	27.62
MDA-MB-468	94.32	71.64	100.93	83.97	26.45	80.31	51.01

Table 2s : Percent growth over 60 cell lines for propyl linker containing derivatives **12h-n**.

Subpanel	12h	12i	12j	12k	12l	12m	12n
**Leukemia**							
CCRF-CEM	88.06	49.05	91.68	82.97	51.91	91.38	70.87
HL-60(TB)	103.91	90.93	107.79	99.78	88.98	110.13	101.05
K-562	79.55	37.18	79.17	73.55	33.90	78.84	67.62
MOLT-4	77.22	33.16	82.28	64.91	38.42	77.01	67.04
RPMI-8226	74.26	57.75	77.95	71.08	**55.65**	78.61	69.69
SR	80.03	36.82	81.45	70.79	57.99	77.57	75.38
**Non-Small Cell Lung Cancer**							
A549/ATCC	97.60	88.76	98.98	95.00	79.03	105.62	86.28
EKVX	88.88	61.18	91.04	81.48	58.10	84.42	75.09
HOP-62	96.87	82.72	101.27	106.86	77.31	94.85	87.09
HOP-92	84.78	87.91	94.63	79.35	56.05	75.78	80.25
NCI-H226	81.00	64.08	83.15	80.23	57.62	73.24	77.83
NCI-H23	91.78	70.37	97.13	92.80	82.49	91.87	91.28
NCI-H322M	109.00	99.49	104.29	102.68	89.91	103.58	103.09
NCI-H460	96.92	79.74	99.81	94.28	79.34	97.26	89.78
NCI-H522	82.70	68.48	82.85	75.53	66.91	81.12	69.98
**Colon Cancer**							
COLO 205	101.58	85.98	109.53	108.12	78.15	108.02	96.44
HCC-2998	106.99	101.61	107.72	111.93	84.77	110.54	112.12
HCT-116	90.34	58.74	94.09	90.27	65.62	89.90	75.93
HCT-15	88.01	82.79	93.09	83.21	53.81	92.30	81.82
HT29	101.35	92.78	103.76	102.98	54.62	99.65	96.35
KM12	71.05	48.89	92.00	68.35	51.71	84.14	79.77
SW-620	107.02	96.22	109.97	106.09	99.55	103.53	106.07
**CNS-Cancer**							
SF-268	91.65	86.29	98.16	91.15	66.50	91.56	86.80
SF-295	92.52	70.60	97.78	86.50	69.77	90.48	81.21
SF-539	89.39	86.21	91.91	85.21	74.49	83.89	87.89
SNB-19	102.61	92.59	104.34	98.60	83.40	100.20	94.18
SNB-75	89.74	82.47	91.68	86.13	65.16	88.25	87.22
U251	98.23	93.81	100.44	96.63	73.24	94.46	90.80
**Melanoma**							
LOX IMVI	91.67	73.96	106.81	94.57	72.31	93.42	85.60
MALME-3M	108.04	86.14	103.48	103.93	93.97	107.43	98.38
M14	----			----			
MDA-MB-435	95.45	64.60	95.52	92.62	72.11	98.74	93.44
SK-MEL-2	108.06	85.79	107.85	104.58	90.97	102.77	97.77
SK-MEL-28	105.29	97.89	108.17	102.23	103.40	105.97	103.48

SK-MEL-5	60.20	2.63	76.22	53.55	29.94	70.07	58.77
UACC-62	84.97	61.64	81.50	77.32	64.07	76.28	62.04
**Ovarian Cancer**							
IGROV1	107.03	91.83	113.55	100.84	74.32	91.45	98.12
OVCAR-3	106.06	86.71	107.13	103.88	81.22	105.16	96.81
OVCAR-4	74.54	44.16	83.02	72.06	52.99	77.41	66.70
OVCAR-5	99.08	98.59	105.12	96.97	87.18	99.62	96.26
OVCAR-8	84.04	62.47	90.84	82.52	59.98	93.70	82.48
NCI/ADR-RES	86.15	64.29	98.20	88.94	55.80	104.95	81.06
SK-OV-3	105.26	85.15	116.51	109.27	80.27	96.21	92.51
**Renal Cancer**							
786-0	98.81	97.67	102.56	91.74	77.21	95.93	91.18
A498	78.36	70.55	72.97	77.20	55.33	81.92	72.14
ACHN	95.68	81.15	94.14	90.38	79.00	88.24	87.13
CAKI-1	87.44	70.98	94.49	84.50	51.38	68.84	60.51
RXF 393	109.91	162.73	123.79	110.60	43.81	80.63	105.26
SN12C	94.31	89.05	91.70	89.16	80.46	92.41	85.91
TK-10	103.41	91.94	104.35	97.80	81.52	103.37	95.71
UO-31	75.30	59.28	87.20	74.26	54.43	67.17	60.73
**Prostate Cancer**							
PC-3	89.19	69.28	92.47	84.69	58.99	89.71	66.68
DU-145	99.24	87.19	100.10	96.50	83.52	100.29	94.49
**Breast Cancer**							
MCF7	87.73	63.70	89.04	86.67	65.52	85.07	83.44
MDA-MB-231/ATCC	92.40	74.42	89.36	83.63	68.70	84.11	83.09
HS 578T	93.67	82.38	105.05	89.95	58.63	84.77	78.28
BT-549	96.55	34.91	90.08	80.80	62.86	92.92	88.17
T-47D	45.80	25.46	65.26	45.29	16.78	66.31	44.68
MDA-MB-468	68.82	50.23	93.79	70.73	52.69	77.17	76.17

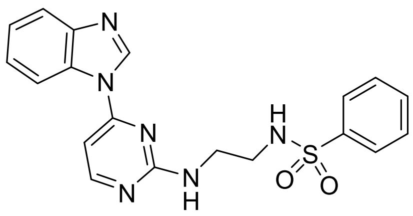

12a

Developmental Therapeutics Program<br>One Dose Mean Graph	NSC: D-828556 / 1	Conc: 1.00E-5 Molar	Test Date: Mar 15, 2021
	Experiment ID: 2103OS92		Report Date: May 06, 2021

Panel/Cell Line	Growth Percent
**Leukemia**	
CCRF-CEM	96.04
HL-60(TB)	112.89
K-562	84.78
MOLT-4	93.72
RPMI-8226	84.93
SR	97.54
**Non-Small Cell Lung Cancer**	
A549/ATCC	103.72
EKVX	91.22
HOP-62	90.71
HOP-92	73.31
NCI-H226	84.57
NCI-H23	96.41
NCI-H322M	94.88
NCI-H460	102.66
NCI-H522	93.85
**Colon Cancer**	
COLO 205	101.30
HCC-2998	105.81
HCT-116	94.63
HCT-15	97.00
HT29	101.92
KM12	99.99
SW-620	112.27
**CNS Cancer**	
SF-268	94.07
SF-295	97.28
SF-539	96.07
SNB-19	99.64
SNB-75	91.75
U251	103.34
**Melanoma**	
LOX IMVI	95.05
MALME-3M	101.04
MDA-MB-435	109.49
SK-MEL-2	114.98
SK-MEL-28	108.05
SK-MEL-5	95.82
UACC-257	107.31
UACC-62	78.31
**Ovarian Cancer**	
IGROV1	89.88
OVCAR-3	108.63
OVCAR-4	93.41
OVCAR-5	92.49
OVCAR-8	98.02
NCI/ADR-RES	104.47
SK-OV-3	101.19
**Renal Cancer**	
786-0	102.76
A498	88.60
ACHN	94.21
CAKI-1	83.72
RXF 393	113.30
SN12C	85.07
TK-10	104.20
UO-31	77.03
**Prostate Cancer**	
PC-3	93.53
DU-145	109.21
**Breast Cancer**	
MCF7	89.32
MDA-MB-231/ATCC	87.62
HS 578T	93.50
BT-549	105.93
T-47D	75.44
MDA-MB-468	94.32
Mean	96.48
Delta	23.17
Range	41.67

Mean Growth Percent - Growth Percent

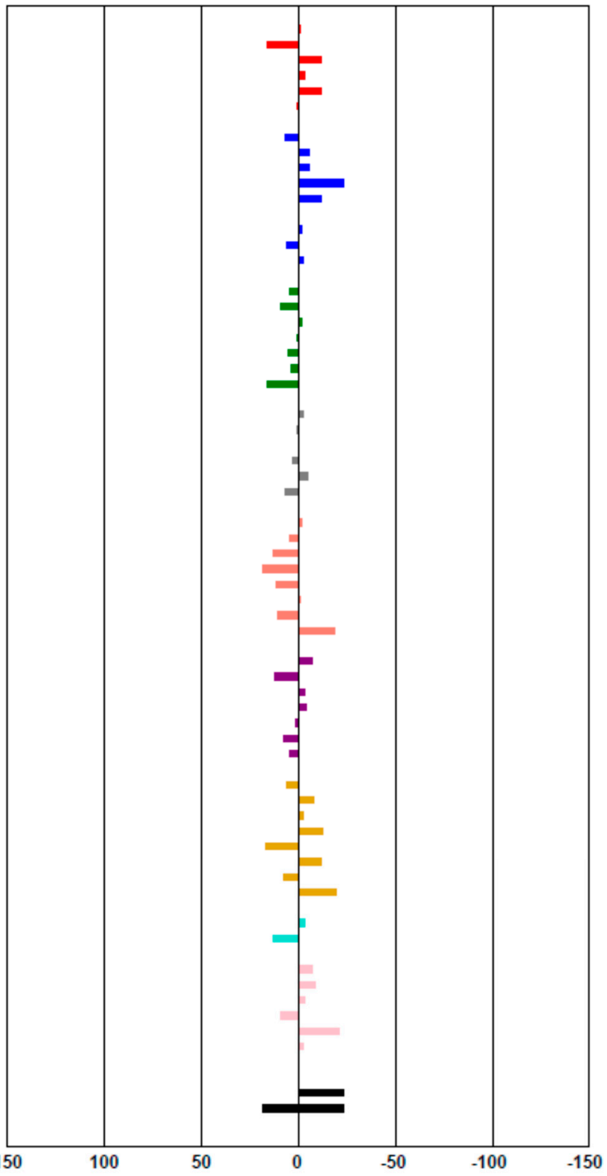

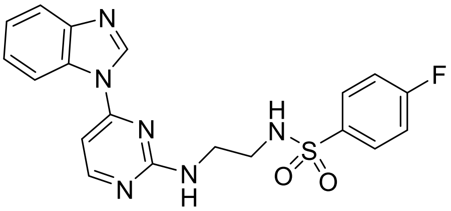

12b

Panel/Cell Line	Growth Percent
**Leukemia**	
CCRF-CEM	93.45
HL-60(TB)	106.15
K-562	84.05
MOLT-4	90.46
RPMI-8226	73.62
SR	85.84
**Non-Small Cell Lung Cancer**	
A549/ATCC	91.26
EKVX	85.24
HOP-62	90.88
HOP-92	82.27
NCI-H226	77.55
NCI-H23	95.81
NCI-H322M	95.62
NCI-H460	96.29
NCI-H522	71.82
**Colon Cancer**	
COLO 205	102.41
HCC-2998	104.40
HCT-116	83.58
HCT-15	100.06
HT29	94.36
KM12	73.43
SW-620	100.16
**CNS Cancer**	
SF-268	88.19
SF-295	93.57
SF-539	83.72
SNB-19	92.97
SNB-75	73.63
U251	99.55
**Melanoma**	
LOX IMVI	93.25
MALME-3M	93.70
MDA-MB-435	97.74
SK-MEL-2	101.47
SK-MEL-28	101.20
SK-MEL-5	78.02
UACC-257	91.58
UACC-62	71.28
**Ovarian Cancer**	
IGROV1	89.26
OVCAR-3	106.17
OVCAR-4	72.29
OVCAR-5	86.58
OVCAR-8	89.01
NCI/ADR-RES	102.88
SK-OV-3	102.17
**Renal Cancer**	
786-0	102.21
A498	80.70
ACHN	88.29
CAKI-1	82.01
RXF 393	105.90
SN12C	89.69
TK-10	97.82
UO-31	72.63
**Prostate Cancer**	
PC-3	91.34
DU-145	91.55
**Breast Cancer**	
MCF7	87.73
MDA-MB-231/ATCC	81.83
HS 578T	85.50
BT-549	82.22
T-47D	64.38
MDA-MB-468	71.64
Mean	89.19
Delta	24.81
Range	41.79

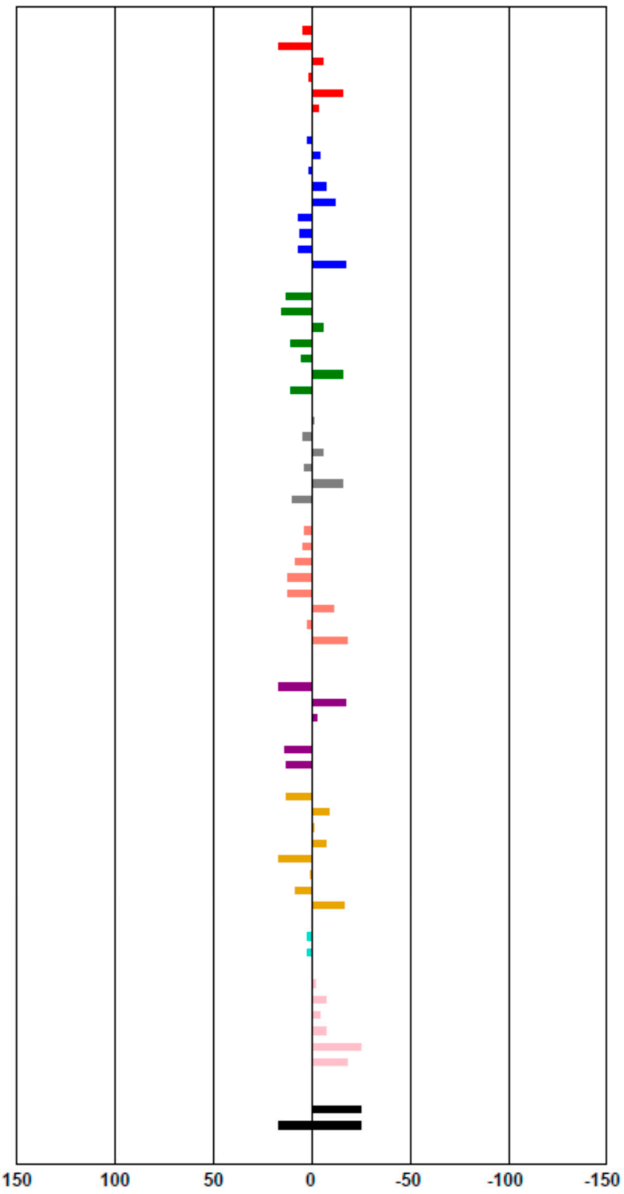

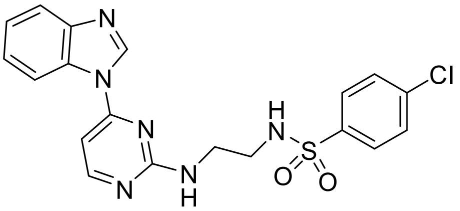

12c

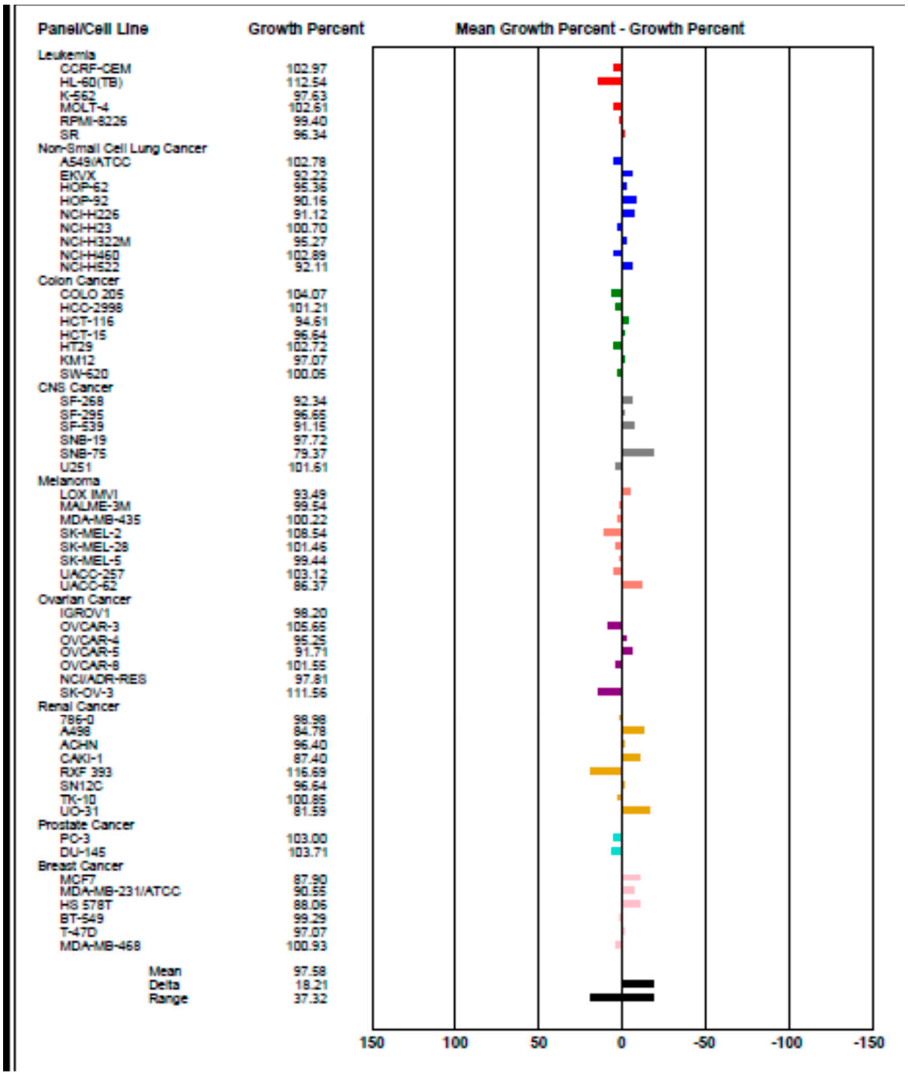
Panel/Cell Line
Growth Percent
Mean Growth Percent - Growth Percent
Leukemia
CCRF-CEM 102.97
HL-60(TB) 112.54
K-562 97.63
MOLT-4 102.61
RPMI-8226 99.40
SR 96.34
Non-Small Cell Lung Cancer
A549/ATCC 102.78
EKVX 92.22
HOP-62 95.36
HOP-92 90.16
NCI-H226 91.12
NCI-H23 100.70
NCI-H322M 95.27
NCI-H460 102.89
NCI-H522 92.11
Colon Cancer
COLO 205 104.07
HCC-2998 101.21
HCT-116 94.61
HCT-15 96.64
HT29 102.72
KM12 97.07
SW-620 100.05
CNS Cancer
SF-268 92.34
SF-295 96.65
SF-539 91.15
SNB-19 97.72
SNB-75 79.37
U251 101.61
Melanoma
LOX IMVI 93.49
MALME-3M 99.54
MDA-MB-435 100.22
SK-MEL-2 108.54
SK-MEL-28 101.46
SK-MEL-5 99.44
UACC-257 103.12
UACC-62 86.37
Ovarian Cancer
IGROV1 98.20
OVCAR-3 105.65
OVCAR-4 95.25
OVCAR-5 91.71
OVCAR-8 101.55
NCI/ADR-RES 97.81
SK-OV-3 111.56
Renal Cancer
786-0 98.98
A498 84.78
ACHN 96.40
CAKI-1 87.40
RXF 393 116.69
SN12C 96.64
TK-10 100.85
UO-31 81.59
Prostate Cancer
PC-3 103.00
DU-145 103.71
Breast Cancer
MCF7 87.90
MDA-MB-231/ATCC 90.55
HS 578T 88.06
BT-549 99.29
T-47D 97.07
MDA-MB-468 100.93
Mean 97.58
Delta 18.21
Range 37.32
150
100
50
0
-50
-100
-150

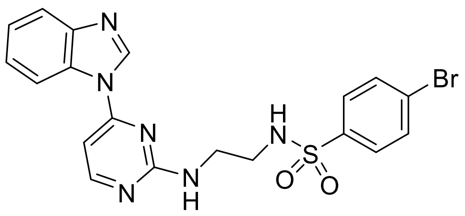

12d

Panel/Cell Line	Growth Percent
Leukemia	
CCRF-CEM	100.92
HL-60(TB)	114.36
K-562	88.40
MOLT-4	95.86
RPMI-8226	79.75
SR	75.08
Non-Small Cell Lung Cancer	
A549/ATCC	100.15
EKVX	83.36
HOP-62	87.17
HOP-92	74.24
NCI-H226	76.53
NCI-H23	97.30
NCI-H322M	107.00
NCI-H460	100.58
NCI-H522	87.18
Colon Cancer	
COLO 205	107.01
HCC-2998	103.48
HCT-116	99.76
HCT-15	95.73
HT29	97.17
KM12	85.44
SW-620	110.11
CNS Cancer	
SF-268	91.24
SF-295	96.48
SF-539	96.18
SNB-19	101.26
SNB-75	74.11
U251	98.60
Melanoma	
LOX IMVI	99.07
MALME-3M	102.82
MDA-MB-435	108.45
SK-MEL-2	107.85
SK-MEL-28	108.16
SK-MEL-5	89.14
UACC-257	108.97
UACC-62	82.77
Ovarian Cancer	
IGROV1	90.72
OVCAR-3	102.75
OVCAR-4	82.22
OVCAR-5	93.95
OVCAR-8	95.60
NCI/ADR-RES	97.63
SK-OV-3	99.94
Renal Cancer	
786-0	102.91
A498	76.27
ACHN	93.63
CAKI-1	81.55
RXF 393	100.99
SN12C	91.48
TK-10	103.12
UO-31	79.80
Prostate Cancer	
PC-3	93.82
DU-145	99.93
Breast Cancer	
MCF7	78.37
MDA-MB-231/ATCC	88.16
HS 578T	85.69
BT-549	115.11
T-47D	59.10
MDA-MB-468	83.97
Mean	93.70
Delta	34.60
Range	56.01

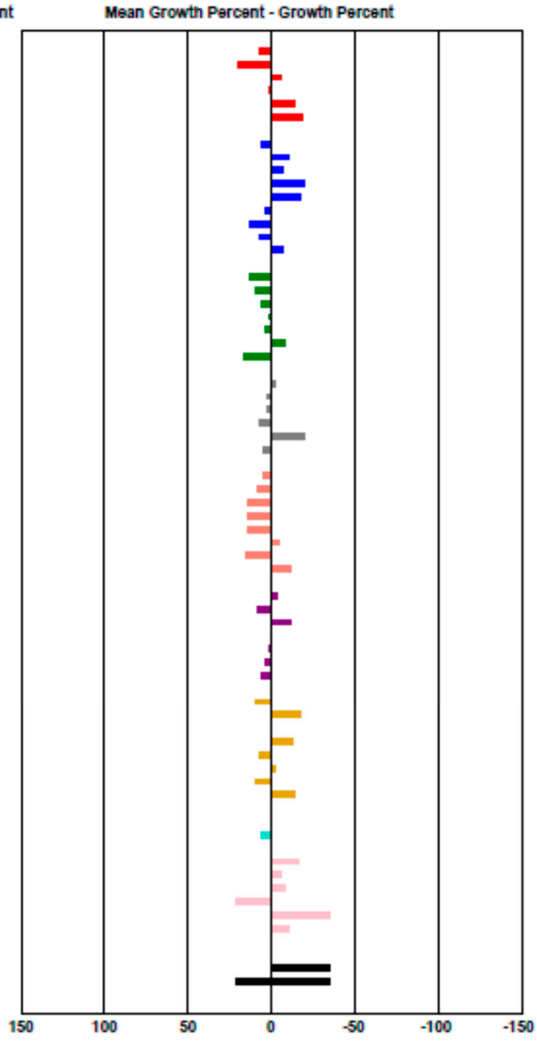

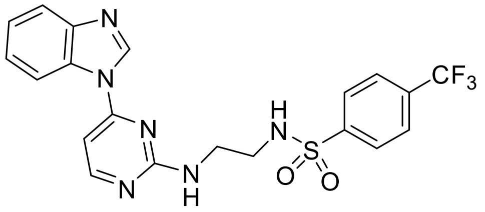

12e

Panel/Cell Line	Growth Percent
Leukemia	
CCRF-CEM	57.64
HL-60(TB)	73.82
K-562	40.82
MOLT-4	45.17
RPMI-8226	44.94
SR	35.52
Non-Small Cell Lung Cancer	
A549/ATCC	55.43
EKVX	34.69
HOP-62	66.07
HOP-92	54.92
NCI-H226	62.71
NCI-H23	54.55
NCI-H322M	85.30
NCI-H460	57.80
NCI-H522	38.17
Colon Cancer	
COLO 205	64.92
HCC-2998	80.46
HCT-116	40.08
HCT-15	60.72
HT29	68.00
KM12	58.72
SW-620	83.49
CNS Cancer	
SF-268	59.50
SF-295	44.68
SF-539	64.84
SNB-19	69.76
SNB-75	46.77
U251	69.60
Melanoma	
LOX IMVI	57.44
MALME-3M	71.37
MDA-MB-435	64.37
SK-MEL-2	67.76
SK-MEL-28	77.19
SK-MEL-5	8.48
UACC-257	36.77
UACC-62	42.41
Ovarian Cancer	
IGROV1	66.39
OVCAR-3	72.90
OVCAR-4	28.42
OVCAR-5	86.24
OVCAR-8	63.50
NCI/ADR-RES	55.91
SK-OV-3	62.23
Renal Cancer	
786-0	69.65
A498	56.61
ACHN	60.80
CAKI-1	43.10
RXF 393	38.25
SN12C	72.54
TK-10	63.40
UO-31	39.85
Prostate Cancer	
PC-3	45.46
DU-145	71.82
Breast Cancer	
MCF7	34.88
MDA-MB-231/ATCC	63.96
HS 578T	42.89
BT-549	53.06
T-47D	22.34
MDA-MB-468	26.45
Mean	55.69
Delta	47.21
Range	77.76

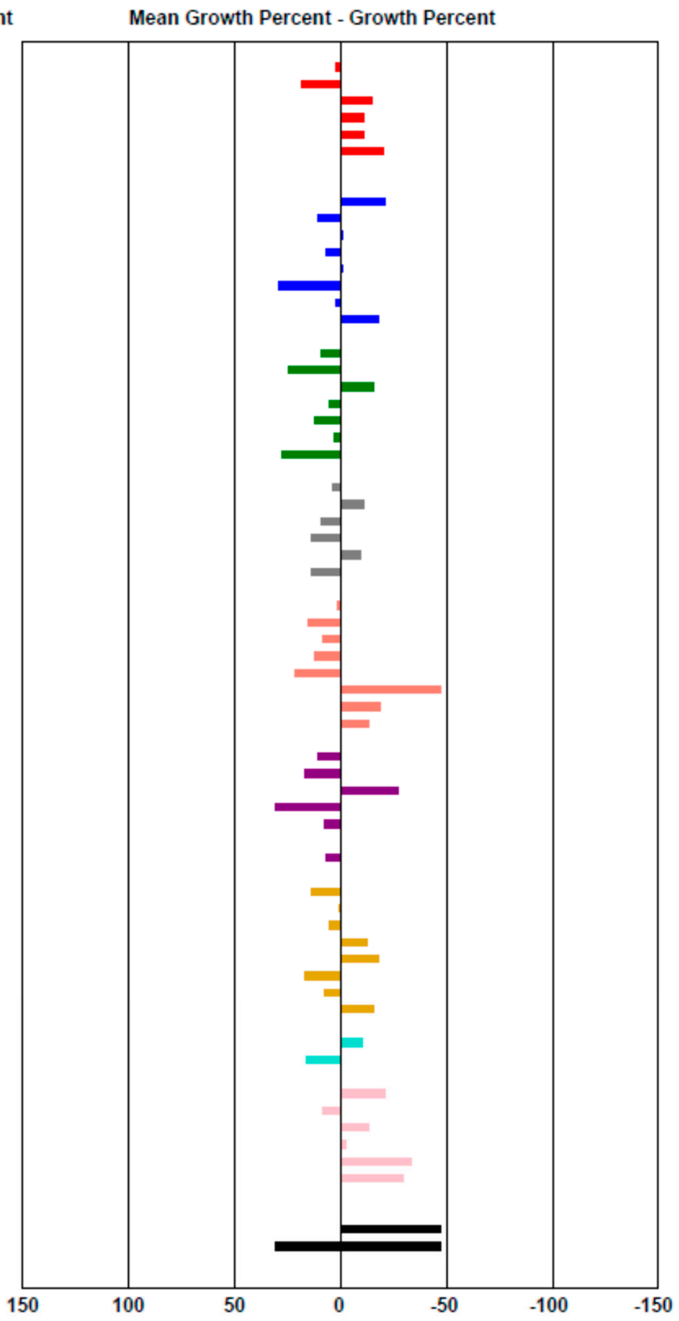

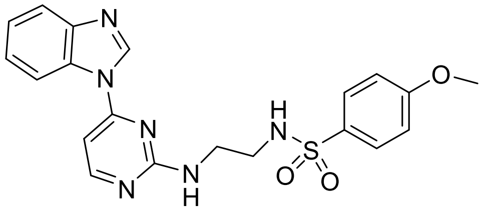

12f

Panel/Cell Line	Growth Percent
**Leukemia**	
CCRF-CEM	102.48
HL-60(TB)	105.97
K-562	92.95
MOLT-4	93.29
RPMI-8226	94.04
SR	89.16
**Non-Small Cell Lung Cancer**	
A549/ATCC	94.62
EKVX	89.19
HOP-62	94.60
HOP-92	75.40
NCI-H226	80.60
NCI-H23	96.92
NCI-H322M	92.58
NCI-H460	106.11
NCI-H522	84.44
**Colon Cancer**	
COLO 205	99.76
HCC-2998	99.72
HCT-116	97.98
HCT-15	93.72
HT29	100.15
KM12	93.20
SW-620	103.20
**CNS Cancer**	
SF-268	86.72
SF-295	96.98
SF-539	96.17
SNB-19	95.30
SNB-75	78.79
U251	102.85
**Melanoma**	
LOX IMVI	91.67
MALME-3M	91.72
MDA-MB-435	96.62
SK-MEL-2	105.98
SK-MEL-28	97.92
SK-MEL-5	92.21
UACC-257	93.51
UACC-62	76.59
**Ovarian Cancer**	
IGROV1	88.99
OVCAR-3	102.87
OVCAR-4	84.28
OVCAR-5	93.88
OVCAR-8	91.47
NCI/ADR-RES	98.74
SK-OV-3	97.45
**Renal Cancer**	
786-0	92.97
A498	82.86
ACHN	97.14
CAKI-1	79.60
RXF 393	94.57
SN12C	88.51
TK-10	92.50
UO-31	77.26
**Prostate Cancer**	
PC-3	97.53
DU-145	99.91
**Breast Cancer**	
MCF7	86.18
MDA-MB-231/ATCC	92.06
HS 578T	84.13
BT-549	97.83
T-47D	82.35
MDA-MB-468	80.31
Mean	92.65
Delta	17.25
Range	30.71

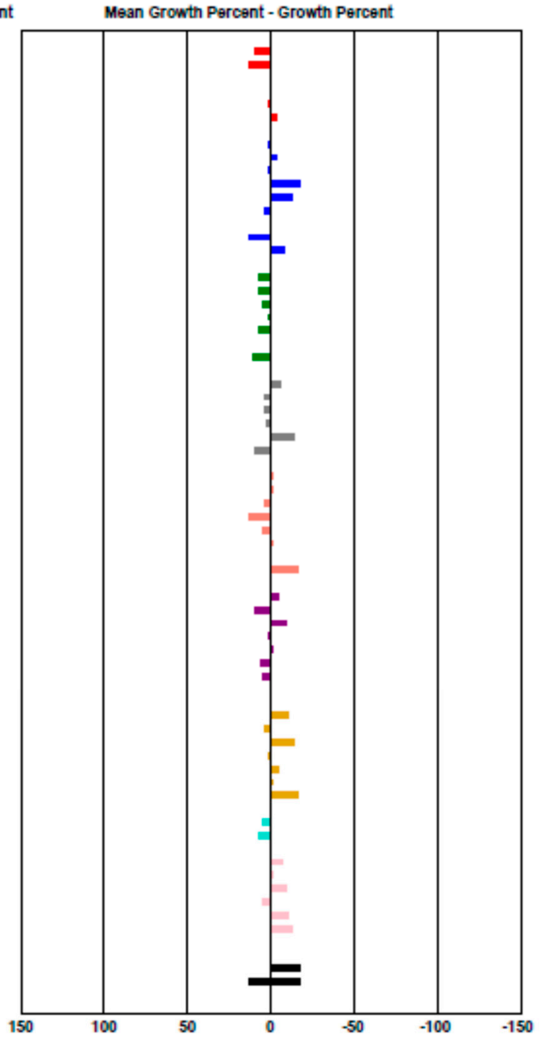

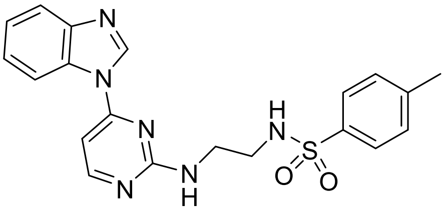

12g

Panel/Cell Line	Growth Percent
**Leukemia**	
CCRF-CEM	69.49
HL-60(TB)	91.22
K-562	56.14
MOLT-4	55.29
RPMI-8226	52.51
SR	53.63
**Non-Small Cell Lung Cancer**	
A549/ATCC	82.36
EKVX	56.10
HOP-62	69.62
HOP-92	57.86
NCI-H226	58.69
NCI-H23	74.63
NCI-H322M	94.38
NCI-H460	79.00
NCI-H522	64.56
**Colon Cancer**	
COLO 205	86.83
HCC-2998	93.09
HCT-116	64.09
HCT-15	80.22
HT29	90.15
KM12	77.40
SW-620	90.84
**CNS Cancer**	
SF-268	68.77
SF-295	75.49
SF-539	79.68
SNB-19	86.81
SNB-75	68.99
U251	85.15
**Melanoma**	
LOX IMVI	75.18
MALME-3M	90.24
MDA-MB-435	84.11
SK-MEL-2	87.74
SK-MEL-28	94.43
SK-MEL-5	20.80
UACC-257	64.19
UACC-62	53.03
**Ovarian Cancer**	
IGROV1	75.18
OVCAR-3	82.02
OVCAR-4	42.15
OVCAR-5	87.83
OVCAR-8	79.83
NCI/ADR-RES	75.74
SK-OV-3	79.46
**Renal Cancer**	
786-0	85.41
A498	62.06
ACHN	72.98
CAKI-1	57.53
RXF 393	55.39
SN12C	79.03
TK-10	83.69
UO-31	52.03
**Prostate Cancer**	
PC-3	76.11
DU-145	89.27
**Breast Cancer**	
MCF7	67.90
MDA-MB-231/ATCC	62.50
HS 578T	64.25
BT-549	63.69
T-47D	27.62
MDA-MB-468	51.01
Mean	71.28
Delta	50.48
Range	73.63

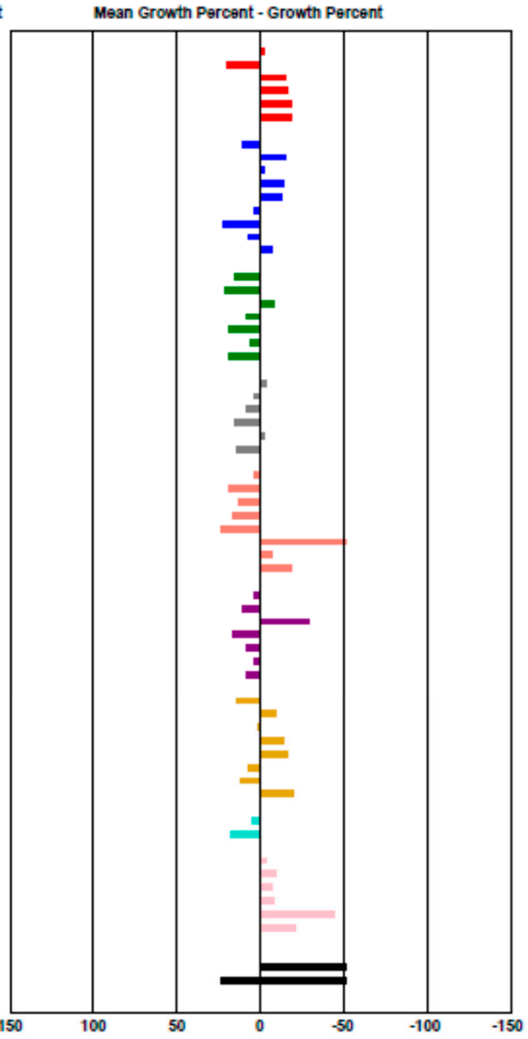

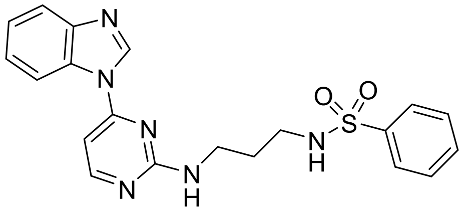

12h

Panel/Cell Line	Growth Percent
Leukemia	
CCRF-CEM	88.06
HL-60(TB)	103.91
K-562	79.55
MOLT-4	77.22
RPMI-8226	74.26
SR	80.03
Non-Small Cell Lung Cancer	
A549/ATCC	97.60
EKVX	88.88
HOP-62	96.87
HOP-92	84.78
NCI-H226	81.00
NCI-H23	91.78
NCI-H322M	109.00
NCI-H460	96.92
NCI-H522	82.70
Colon Cancer	
COLO 205	101.58
HCC-2998	106.99
HCT-116	90.34
HCT-15	88.01
HT29	101.35
KM12	71.05
SW-620	107.02
CNS Cancer	
SF-268	91.65
SF-295	92.52
SF-539	89.39
SNB-19	102.61
SNB-75	89.74
U251	98.23
Melanoma	
LOX IMVI	91.67
MALME-3M	108.04
MDA-MB-435	95.45
SK-MEL-2	108.06
SK-MEL-28	105.29
SK-MEL-5	60.20
UACC-257	103.56
UACC-62	84.97
Ovarian Cancer	
IGROV1	107.03
OVCAR-3	106.06
OVCAR-4	74.54
OVCAR-5	99.08
OVCAR-8	84.04
NCI/ADR-RES	86.15
SK-OV-3	105.26
Renal Cancer	
786-0	98.81
A498	78.36
ACHN	95.68
CAKI-1	87.44
RXF 393	109.91
SN12C	94.31
TK-10	103.41
UO-31	75.30
Prostate Cancer	
PC-3	89.19
DU-145	99.24
Breast Cancer	
MCF7	87.73
MDA-MB-231/ATCC	92.40
HS 578T	93.67
BT-549	96.55
T-47D	45.80
MDA-MB-468	68.82
Mean	91.51
Delta	45.71
Range	64.11

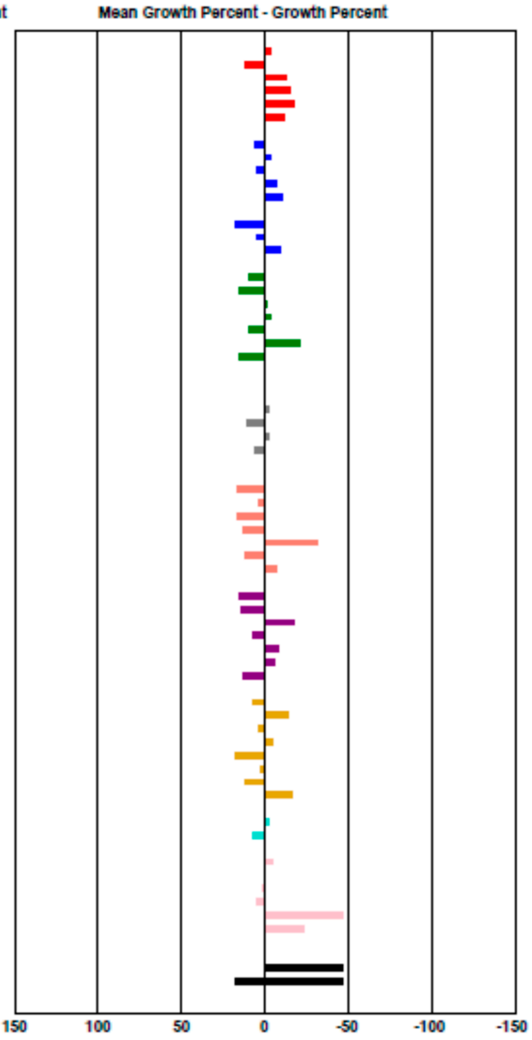

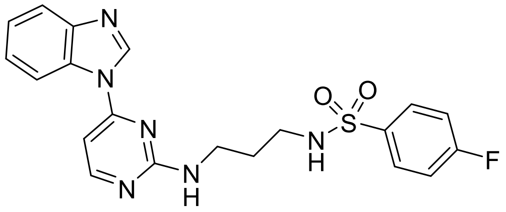

12i

Panel/Cell Line	Growth Percent
Leukemia	
CCRF-CEM	49.05
HL-60(TB)	90.93
K-562	37.18
MOLT-4	33.16
RPMI-8226	57.75
SR	36.82
Non-Small Cell Lung Cancer	
A549/ATCC	88.76
EKVX	61.18
HOP-62	82.72
HOP-92	87.91
NCI-H226	64.08
NCI-H23	70.37
NCI-H322M	99.49
NCI-H460	79.74
NCI-H522	68.48
Colon Cancer	
COLO 205	85.98
HCC-2998	101.61
HCT-116	58.74
HCT-15	82.79
HT29	92.78
KM12	48.89
SW-620	96.22
CNS Cancer	
SF-268	86.29
SF-295	70.60
SF-539	86.21
SNB-19	92.59
SNB-75	82.47
U251	93.81
Melanoma	
LOX IMVI	73.96
MALME-3M	86.14
MDA-MB-435	64.60
SK-MEL-2	85.79
SK-MEL-28	97.89
SK-MEL-5	2.63
UACC-257	66.21
UACC-62	61.64
Ovarian Cancer	
IGROV1	91.83
OVCAR-3	86.71
OVCAR-4	44.16
OVCAR-5	98.59
OVCAR-8	62.47
NCI/ADR-RES	64.29
SK-OV-3	85.15
Renal Cancer	
786-0	97.67
A498	70.55
ACHN	81.15
CAKI-1	70.98
RXF 393	162.73
SN12C	89.05
TK-10	91.94
UO-31	59.28
Prostate Cancer	
PC-3	69.28
DU-145	87.19
Breast Cancer	
MCF7	63.70
MDA-MB-231/ATCC	74.42
HS 578T	82.38
BT-549	34.91
T-47D	25.46
MDA-MB-468	50.23
Mean	74.06
Delta	71.43
Range	160.10

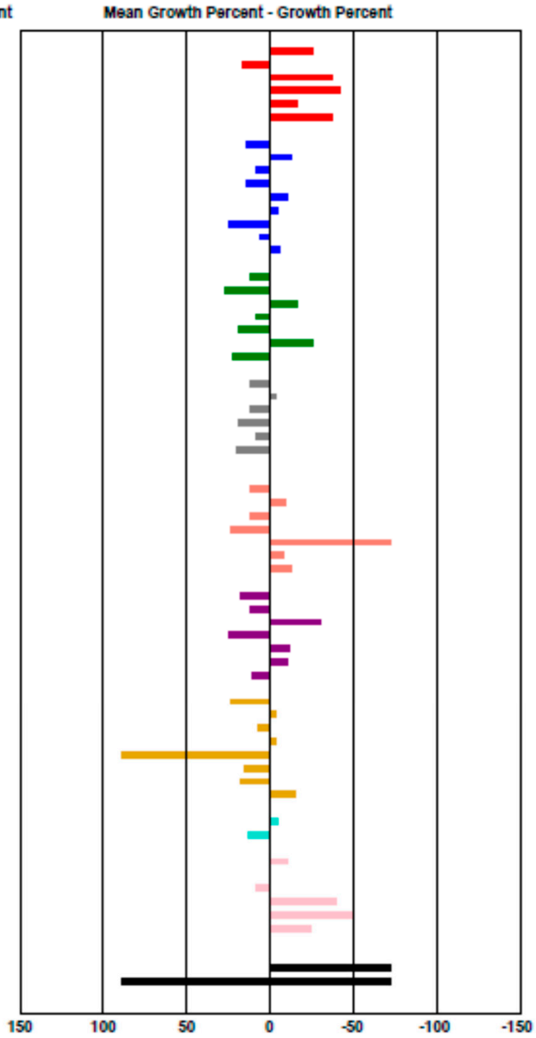

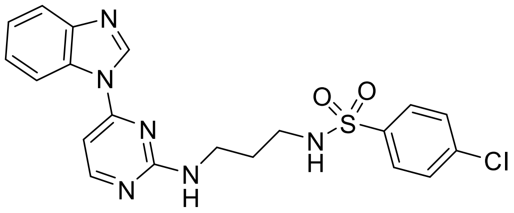

12j

Panel/Cell Line	Growth Percent
Leukemia	
CCRF-CEM	91.68
HL-60(TB)	107.79
K-562	79.17
MOLT-4	82.28
RPMI-8226	77.95
SR	81.45
Non-Small Cell Lung Cancer	
A549/ATCC	98.98
EKVX	91.04
HOP-62	101.27
HOP-92	94.63
NCI-H226	83.15
NCI-H23	97.13
NCI-H322M	104.29
NCI-H460	99.81
NCI-H522	82.85
Colon Cancer	
COLO 205	109.53
HCC-2998	107.72
HCT-116	94.09
HCT-15	93.09
HT29	103.76
KM12	92.00
SW-620	109.97
CNS Cancer	
SF-268	98.16
SF-295	97.78
SF-539	91.91
SNB-19	104.34
SNB-75	91.68
U251	100.44
Melanoma	
LOX IMVI	106.81
MALME-3M	103.48
MDA-MB-435	95.52
SK-MEL-2	107.85
SK-MEL-28	108.17
SK-MEL-5	76.22
UACC-257	106.73
UACC-62	81.50
Ovarian Cancer	
IGROV1	113.55
OVCAR-3	107.13
OVCAR-4	83.02
OVCAR-5	105.12
OVCAR-8	90.84
NCI/ADR-RES	98.20
SK-OV-3	116.51
Renal Cancer	
786-0	102.56
A498	72.97
ACHN	94.14
CAKI-1	94.49
RXF 393	123.79
SN12C	91.70
TK-10	104.35
UO-31	87.20
Prostate Cancer	
PC-3	92.47
DU-145	100.10
Breast Cancer	
MCF7	89.04
MDA-MB-231/ATCC	89.36
HS 578T	105.05
BT-549	90.08
T-47D	65.26
MDA-MB-468	93.79
Mean	96.02
Delta	30.76
Range	58.53

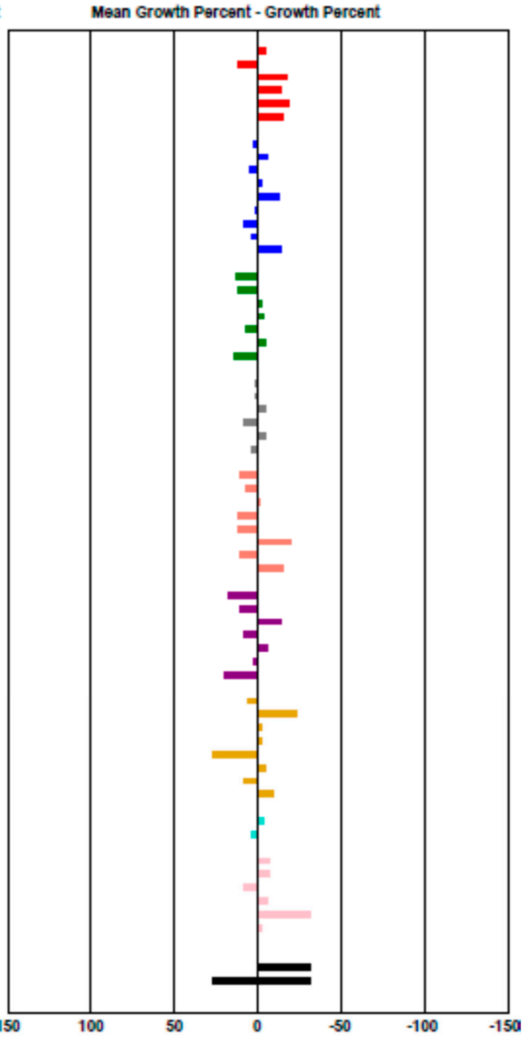

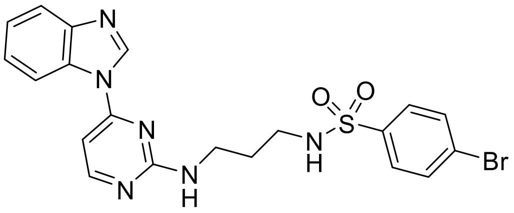

12k

Panel/Cell Line	Growth Percent
Leukemia	
CCRF-CEM	82.97
HL-60(TB)	99.78
K-562	73.55
MOLT-4	64.91
RPMI-8226	71.08
SR	70.79
Non-Small Cell Lung Cancer	
A549/ATCC	95.00
EKVX	81.48
HOP-62	106.86
HOP-92	79.35
NCI-H226	80.23
NCI-H23	92.80
NCI-H322M	102.68
NCI-H460	94.28
NCI-H522	75.53
Colon Cancer	
COLO 205	108.12
HCC-2998	111.93
HCT-116	90.27
HCT-15	83.21
HT29	102.98
KM12	68.35
SW-620	106.09
CNS Cancer	
SF-268	91.15
SF-295	86.50
SF-539	85.21
SNB-19	98.60
SNB-75	86.13
U251	96.63
Melanoma	
LOX IMVI	94.57
MALME-3M	103.93
MDA-MB-435	92.62
SK-MEL-2	104.58
SK-MEL-28	102.23
SK-MEL-5	53.55
UACC-257	100.12
UACC-62	77.32
Ovarian Cancer	
IGROV1	100.84
OVCAR-3	103.88
OVCAR-4	72.06
OVCAR-5	96.97
OVCAR-8	82.52
NCI/ADR-RES	88.94
SK-OV-3	109.27
Renal Cancer	
786-0	91.74
A498	77.20
ACHN	90.38
CAKI-1	84.50
RXF 393	110.60
SN12C	89.16
TK-10	97.80
UO-31	74.26
Prostate Cancer	
PC-3	84.69
DU-145	96.50
Breast Cancer	
MCF7	86.67
MDA-MB-231/ATCC	83.63
HS 578T	89.95
BT-549	80.80
T-47D	45.29
MDA-MB-468	70.73
Mean	88.54
Delta	43.25
Range	66.64

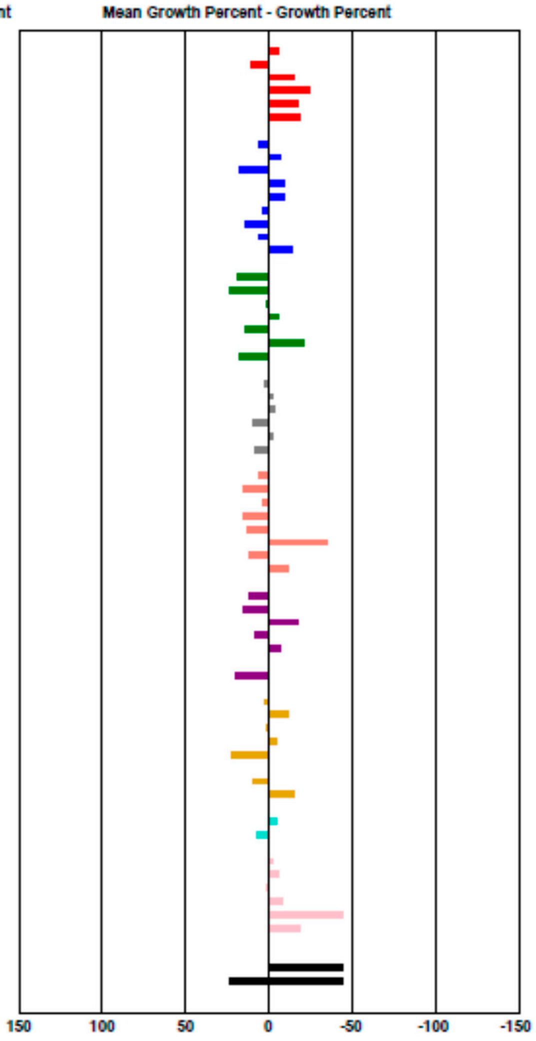

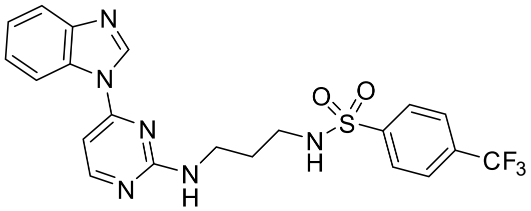

12l

Panel/Cell Line	Growth Percent
**Leukemia**	
CCRF-CEM	70.87
HL-60(TB)	101.05
K-562	67.62
MOLT-4	67.04
RPMI-8226	69.69
SR	75.38
**Non-Small Cell Lung Cancer**	
A549/ATCC	86.28
EKVX	75.09
HOP-62	87.09
HOP-92	80.25
NCI-H226	77.83
NCI-H23	91.28
NCI-H322M	103.09
NCI-H460	89.78
NCI-H522	69.98
**Colon Cancer**	
COLO 205	96.44
HCC-2998	112.12
HCT-116	75.93
HCT-15	81.82
HT29	96.35
KM12	79.77
SW-620	106.07
**CNS Cancer**	
SF-268	86.80
SF-295	81.21
SF-539	87.89
SNB-19	94.18
SNB-75	87.22
U251	90.80
**Melanoma**	
LOX IMVI	85.60
MALME-3M	98.38
MDA-MB-435	93.44
SK-MEL-2	97.77
SK-MEL-28	103.48
SK-MEL-5	58.77
UACC-257	97.76
UACC-62	62.04
**Ovarian Cancer**	
IGROV1	98.12
OVCAR-3	96.81
OVCAR-4	66.70
OVCAR-5	96.26
OVCAR-8	82.48
NCI/ADR-RES	81.06
SK-OV-3	92.51
**Renal Cancer**	
786-0	91.18
A498	72.14
ACHN	87.13
CAKI-1	60.51
RXF 393	105.26
SN12C	85.91
TK-10	95.71
UO-31	60.73
**Prostate Cancer**	
PC-3	66.68
DU-145	94.49
**Breast Cancer**	
MCF7	83.44
MDA-MB-231/ATCC	83.09
HS 578T	78.28
BT-549	88.17
T-47D	44.68
MDA-MB-468	76.17
Mean	84.30
Delta	39.62
Range	67.44

Mean Growth Percent - Growth Percent

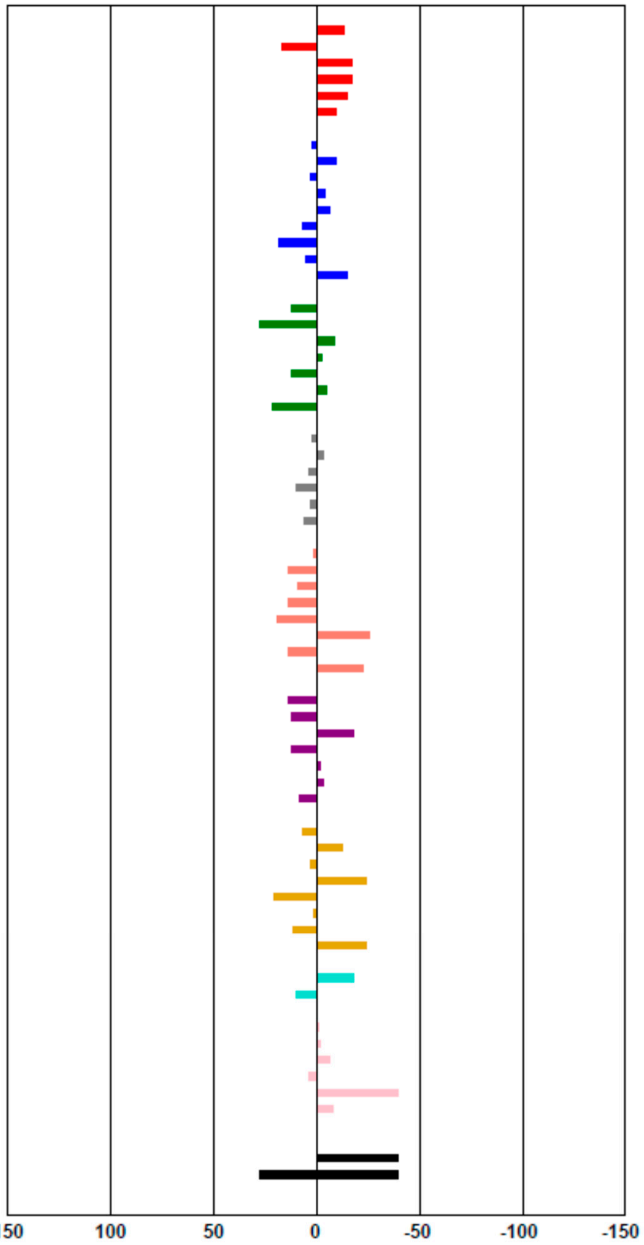

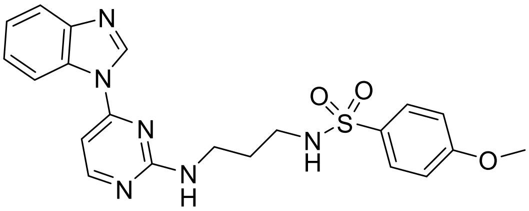

12m

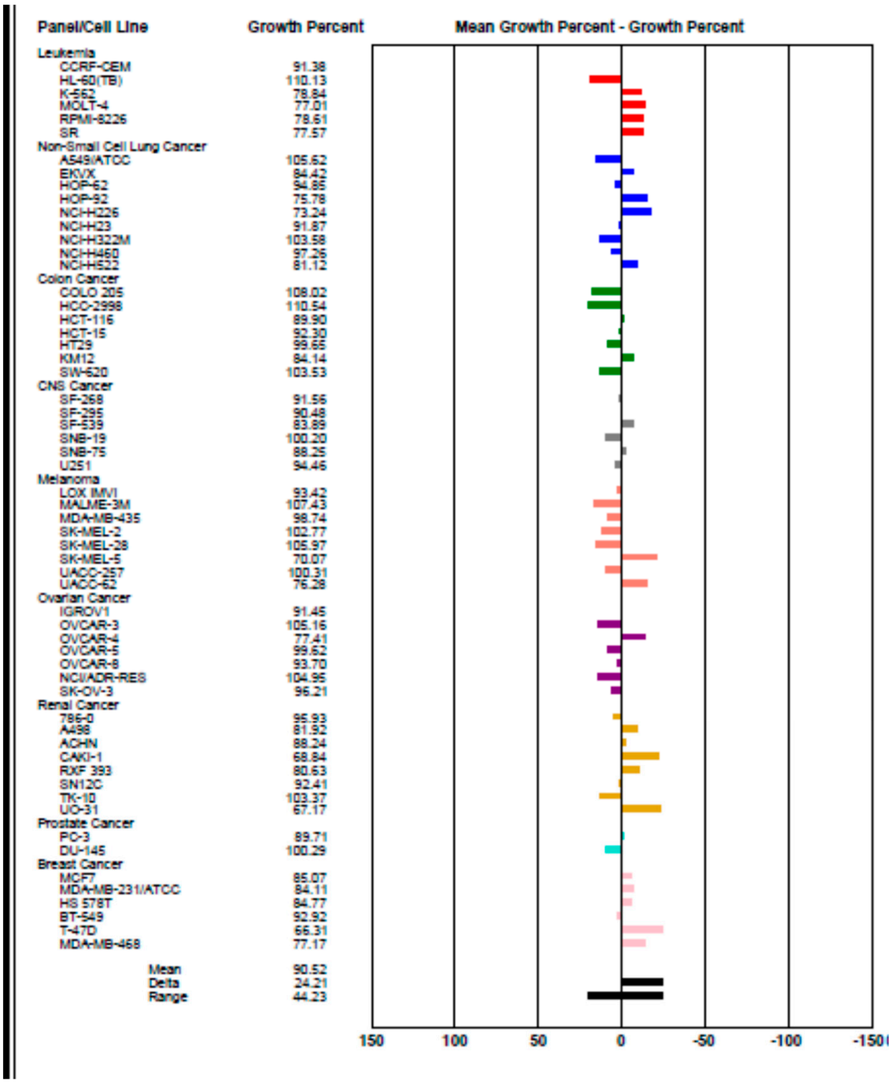
Panel/Cell Line
Growth Percent
Mean Growth Percent - Growth Percent
Leukemia
CCRF-CEM 91.38
HL-60(TB) 110.13
K-562 78.84
MOLT-4 77.01
RPMI-8226 78.61
SR 77.57
Non-Small Cell Lung Cancer
A549/ATCC 105.62
EKVX 84.42
HOP-62 94.85
HOP-92 75.78
NCI-H226 73.24
NCI-H23 91.87
NCI-H322M 103.58
NCI-H460 97.26
NCI-H522 81.12
Colon Cancer
COLO 205 108.02
HCC-2998 110.54
HCT-116 89.90
HCT-15 92.30
HT29 99.65
KM12 84.14
SW-620 103.53
CNS Cancer
SF-268 91.56
SF-295 90.48
SF-539 83.89
SNB-19 100.20
SNB-75 88.25
U251 94.46
Melanoma
LOX IMVI 93.42
MALME-3M 107.43
MDA-MB-435 98.74
SK-MEL-2 102.77
SK-MEL-28 105.97
SK-MEL-5 70.07
UACC-257 100.31
UACC-62 76.28
Ovarian Cancer
IGROV1 91.45
OVCAR-3 105.16
OVCAR-4 77.41
OVCAR-5 99.62
OVCAR-8 93.70
NCI/ADR-RES 104.95
SK-OV-3 96.21
Renal Cancer
786-0 95.93
A498 81.92
ACHN 88.24
CAKI-1 68.84
RXF 393 80.63
SN12C 92.41
TK-10 103.37
UO-31 67.17
Prostate Cancer
PC-3 89.71
DU-145 100.29
Breast Cancer
MCF7 85.07
MDA-MB-231/ATCC 84.11
HS 578T 84.77
BT-549 92.92
T-47D 66.31
MDA-MB-468 77.17
Mean 90.52
Delta 24.21
Range 44.23
150
100
50
0
-50
-100
-150

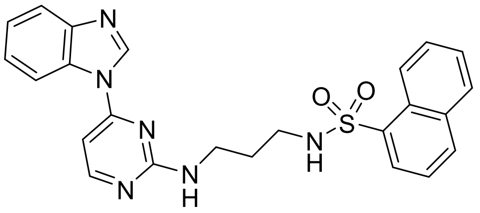

12n

Panel/Cell Line	Growth Percent
Leukemia	
CCRF-CEM	83.74
HL-60(TB)	108.47
K-562	80.28
MOLT-4	80.14
RPMI-8226	74.27
SR	74.04
Non-Small Cell Lung Cancer	
A549/ATCC	100.79
EKVX	92.90
HOP-62	94.45
HOP-92	87.57
NCI-H226	82.90
NCI-H23	98.60
NCI-H322M	123.97
NCI-H460	99.38
NCI-H522	79.35
Colon Cancer	
COLO 205	97.22
HCC-2998	117.25
HCT-116	94.07
HCT-15	90.32
HT29	110.72
KM12	86.98
SW-620	100.09
CNS Cancer	
SF-268	96.50
SF-295	92.50
SF-539	90.60
SNB-19	100.11
SNB-75	69.99
U251	97.07
Melanoma	
LOX IMVI	94.13
MALME-3M	106.29
MDA-MB-435	92.55
SK-MEL-2	103.93
SK-MEL-28	101.45
SK-MEL-5	72.11
UACC-257	103.32
UACC-62	78.50
Ovarian Cancer	
IGROV1	108.62
OVCAR-3	110.69
OVCAR-4	88.10
OVCAR-5	98.24
OVCAR-8	85.03
NCI/ADR-RES	93.57
SK-OV-3	105.91
Renal Cancer	
786-0	107.09
A498	80.22
ACHN	94.50
CAKI-1	81.26
RXF 393	112.51
SN12C	92.65
TK-10	105.02
UO-31	94.21
Prostate Cancer	
PC-3	89.39
DU-145	105.61
Breast Cancer	
MCF7	93.33
MDA-MB-231/ATCC	84.00
HS 578T	83.60
BT-549	94.98
T-47D	56.64
MDA-MB-468	88.95
Mean	93.40
Delta	36.76
Range	67.33

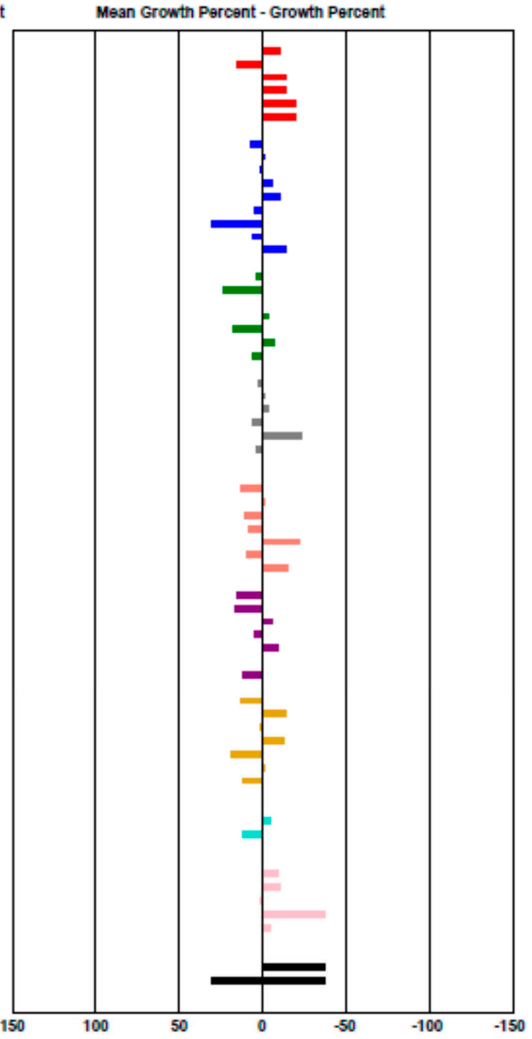

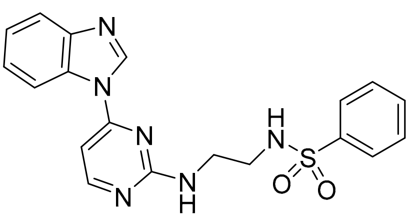

12a

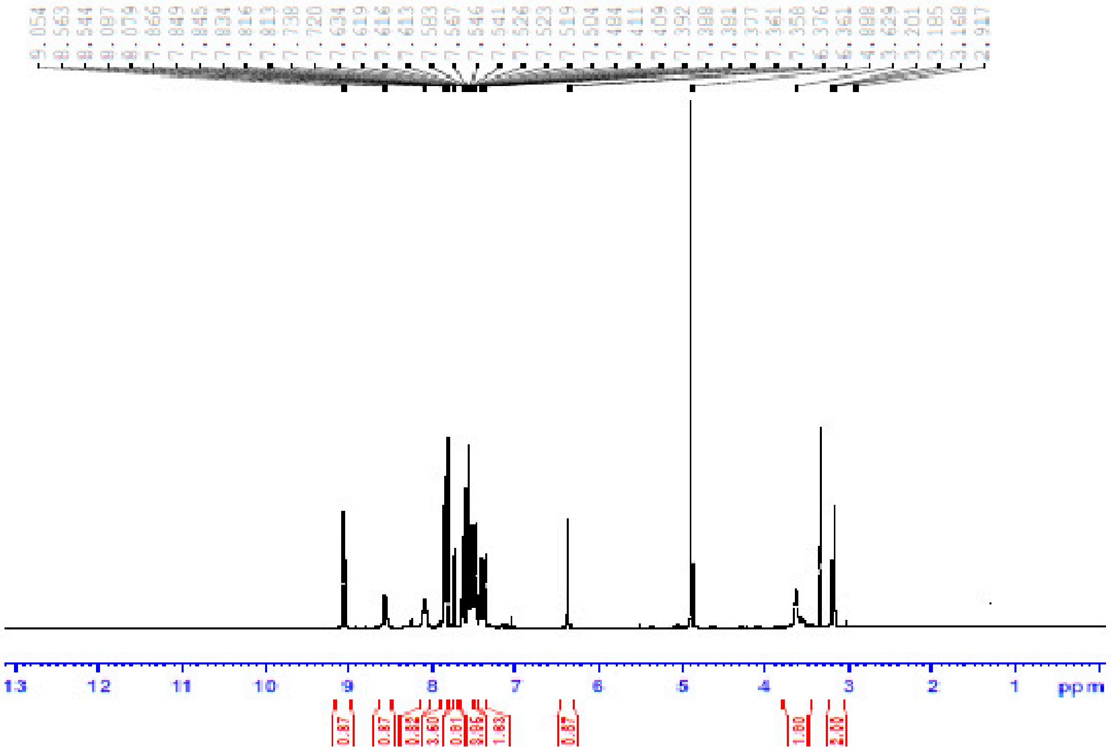

13
12
11
10
9
8
7
6
5
4
3
2
1
ppm
0.87
0.87
0.88
3.60
0.81
3.96
1.83
0.87
1.80
2.00

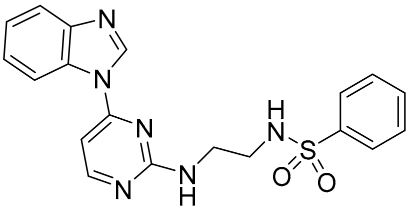

23s

12a

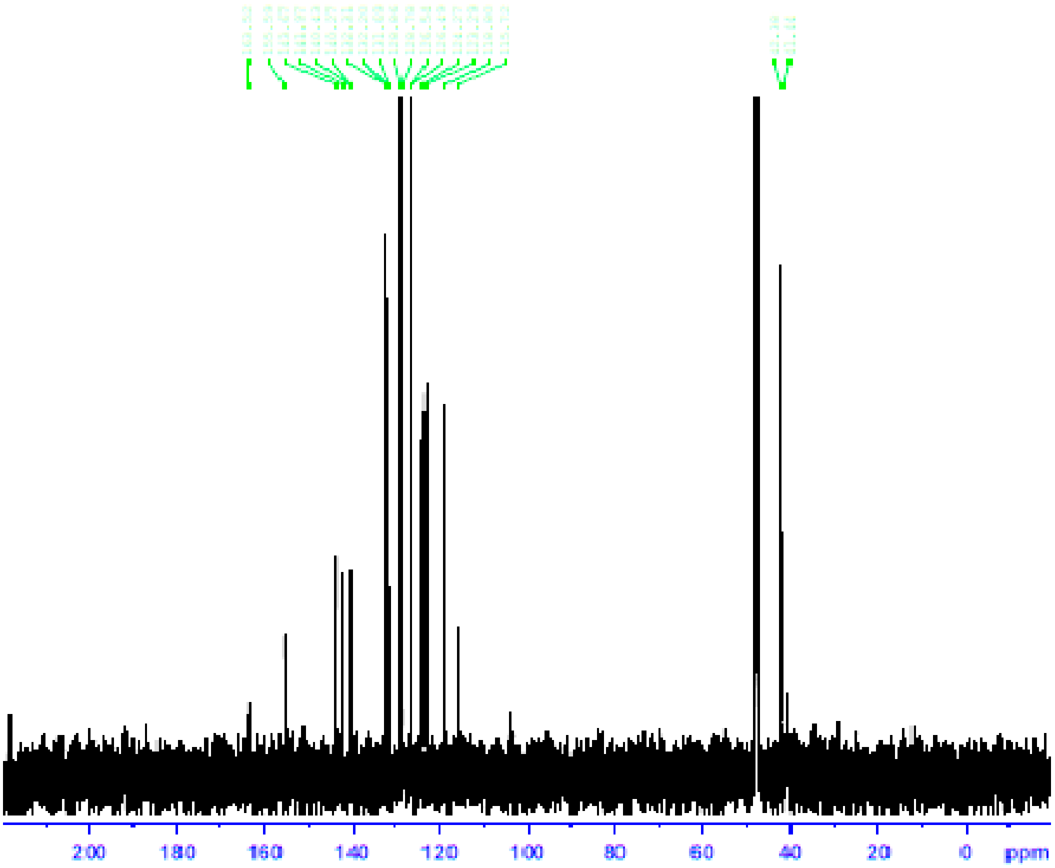

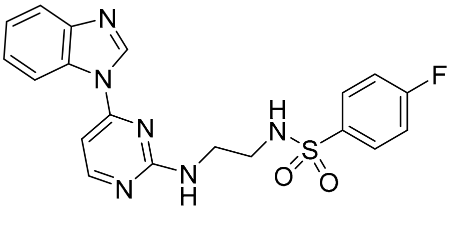

12b

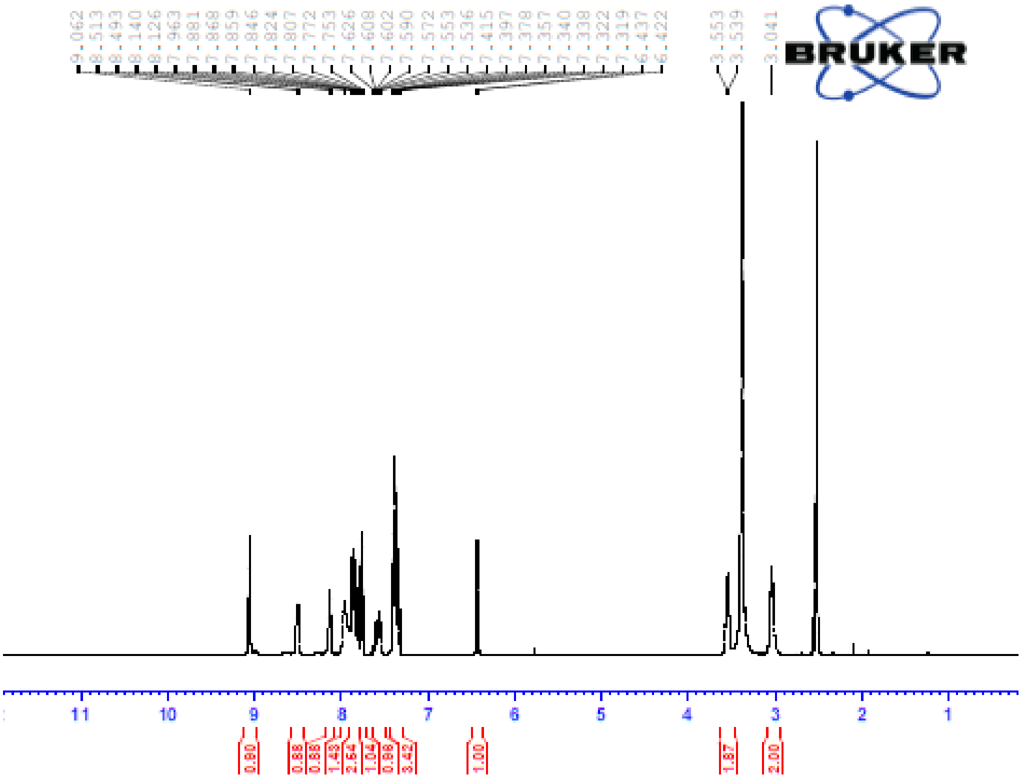
9.062
8.513
8.493
8.140
8.126
7.963
7.881
7.868
7.859
7.846
7.824
7.807
7.772
7.753
7.626
7.608
7.602
7.590
7.572
7.553
7.536
7.415
7.397
7.378
7.357
7.340
7.338
7.322
7.319
6.437
6.422
3.553
3.539
3.041
BRUKER
11
10
9
8
7
6
5
4
3
2
1
0.80
0.88
0.88
1.43
2.64
1.04
0.98
3.42
1.00
1.87
2.00

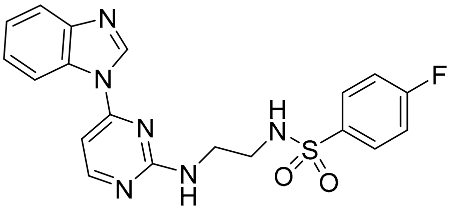
N
N
F
H
N
N
S
O
O
N
H

12b

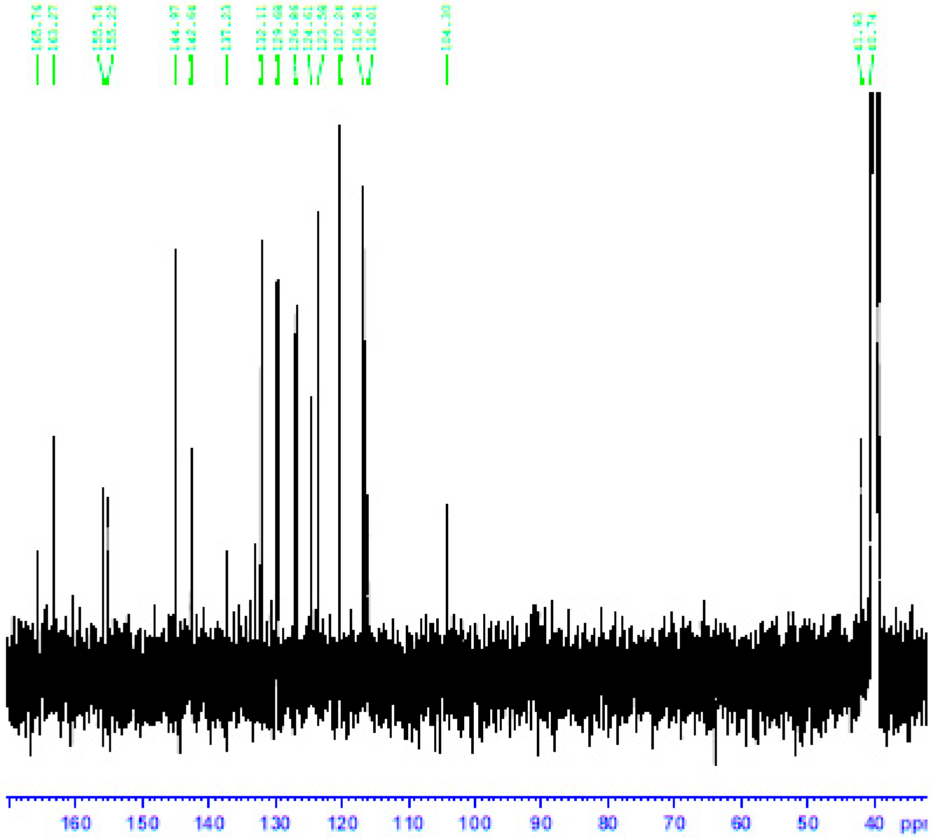

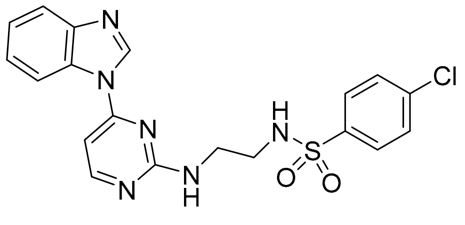

12c

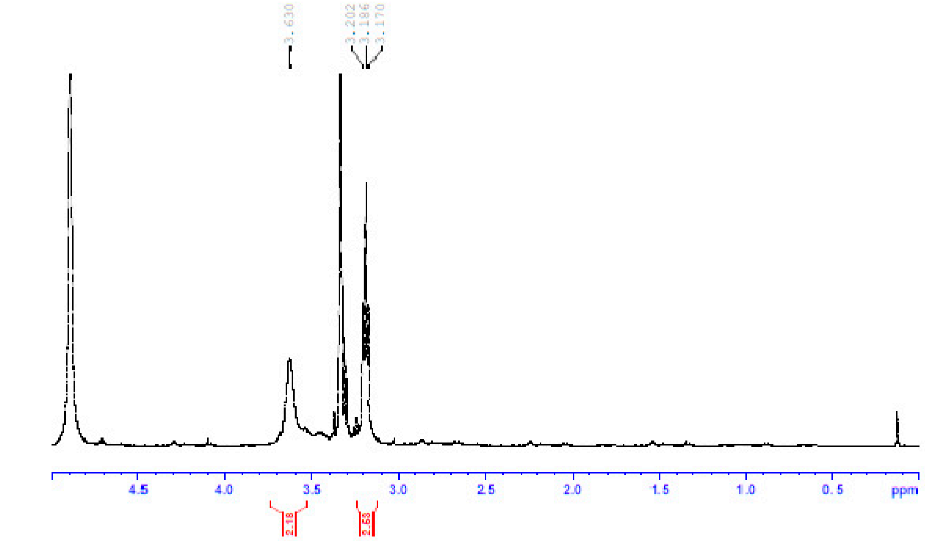
3.630
3.202
3.186
3.170
4.5
4.0
3.5
3.0
2.5
2.0
1.5
1.0
0.5
ppm
2.18
2.58

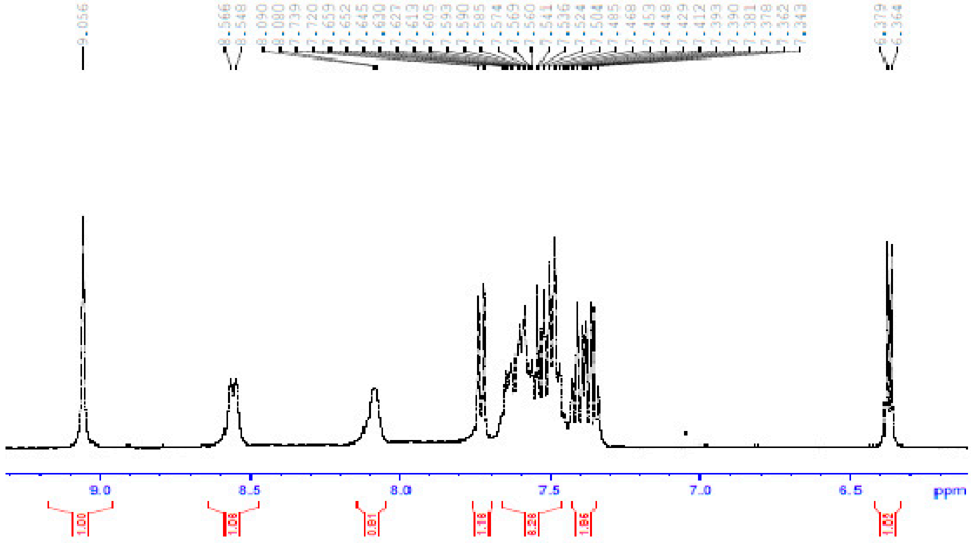
9.056
8.566
8.548
6.379
6.364
9.0
8.5
8.0
7.5
7.0
6.5
ppm
1.00
1.08
0.81
1.18
1.86

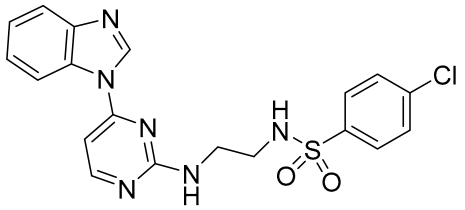
N
N
N
N
N
H
H
N
S
O
O
Cl

12c

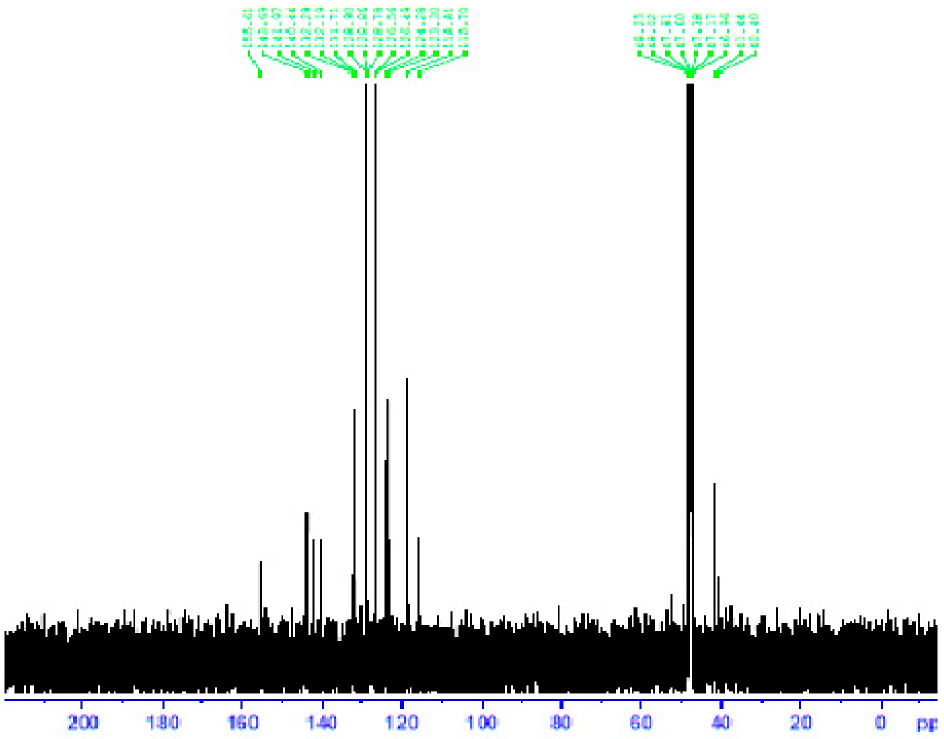

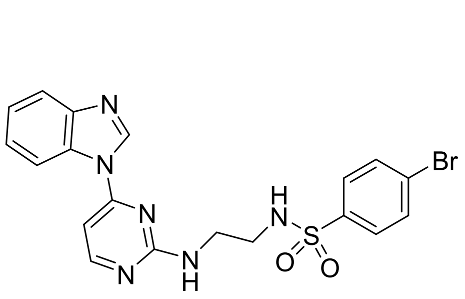

12d

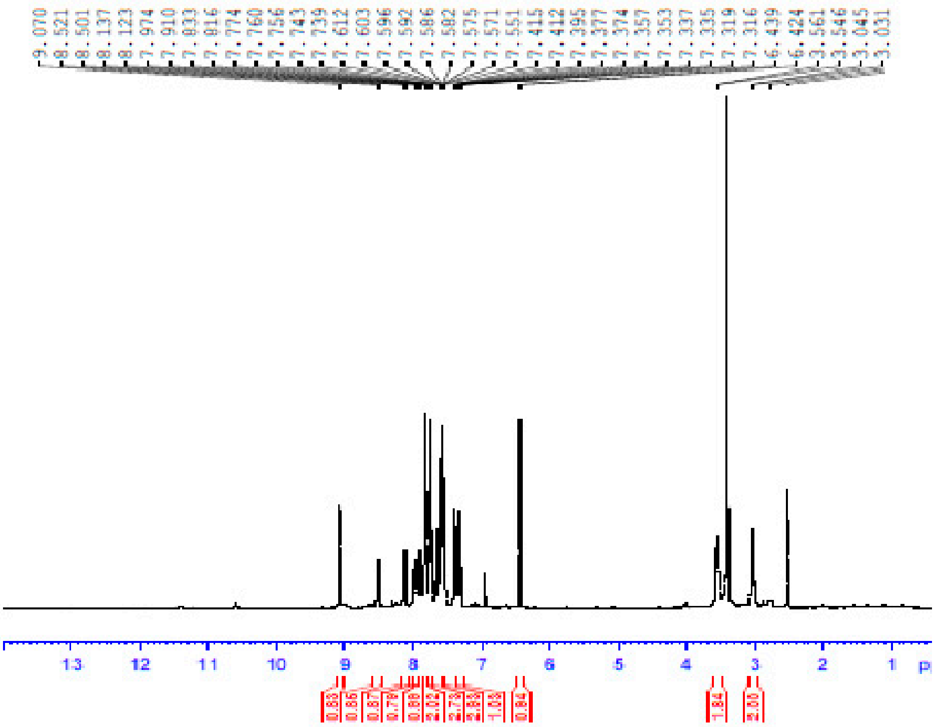

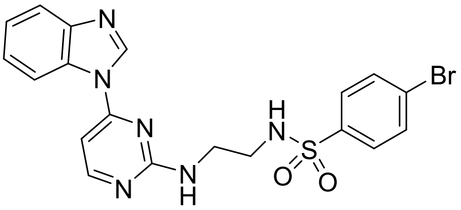

12d

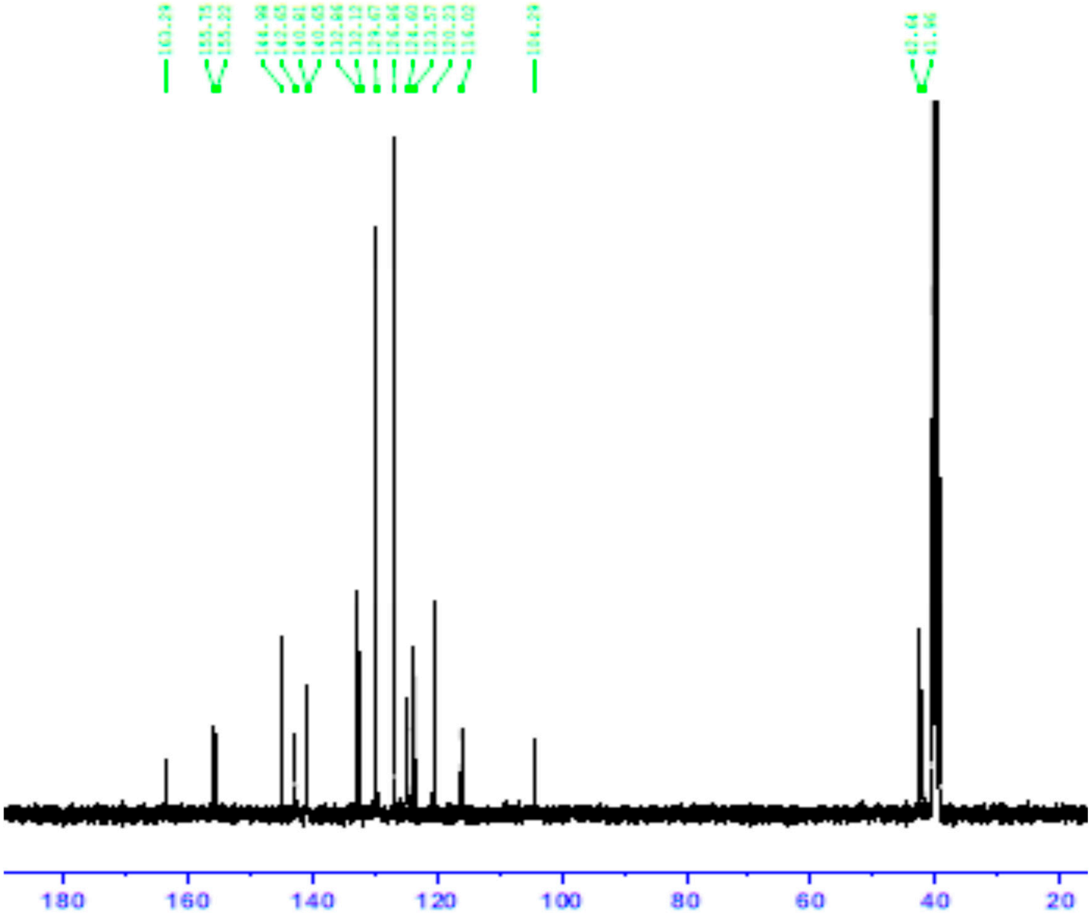

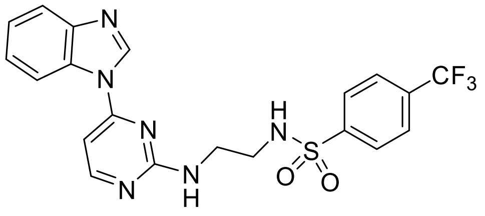

12e

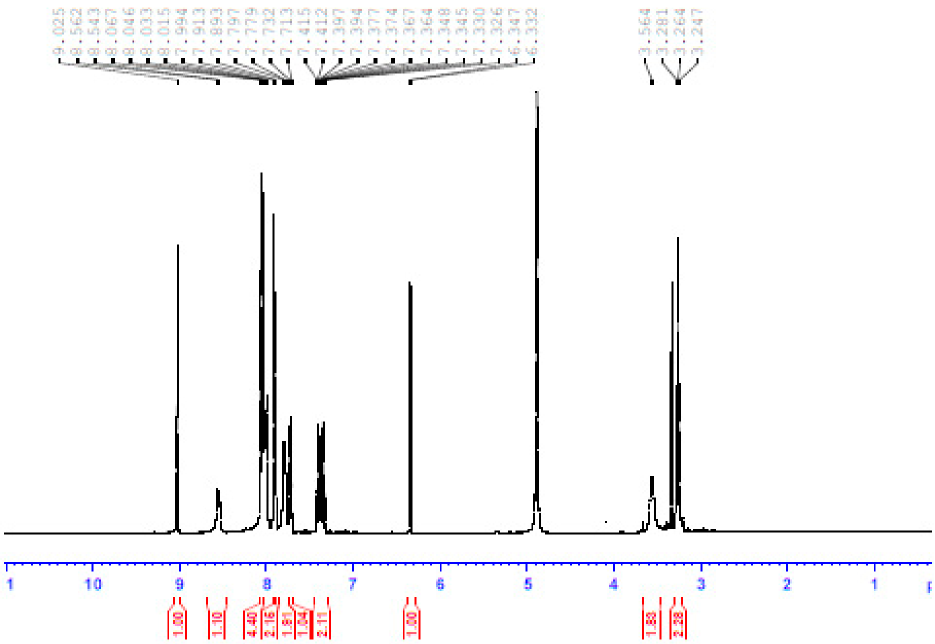

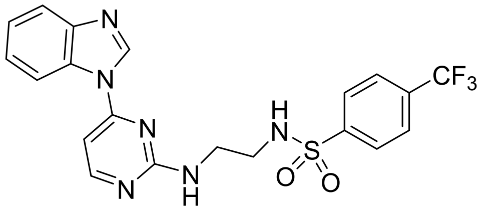

31s

12e

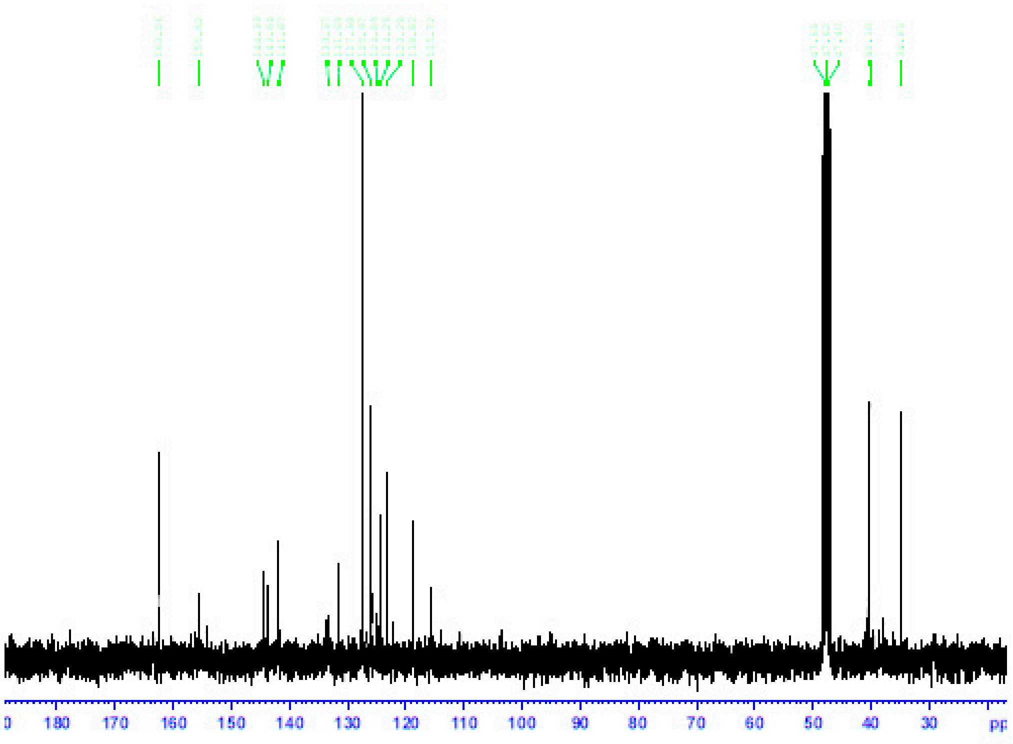

180
170
160
150
140
130
120
110
100
90
80
70
60
50
40
30
pp

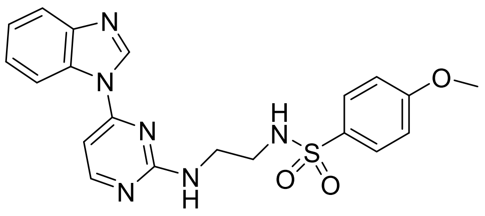

N
N
N
N
H
N
H
N
S
O
O
O

12f

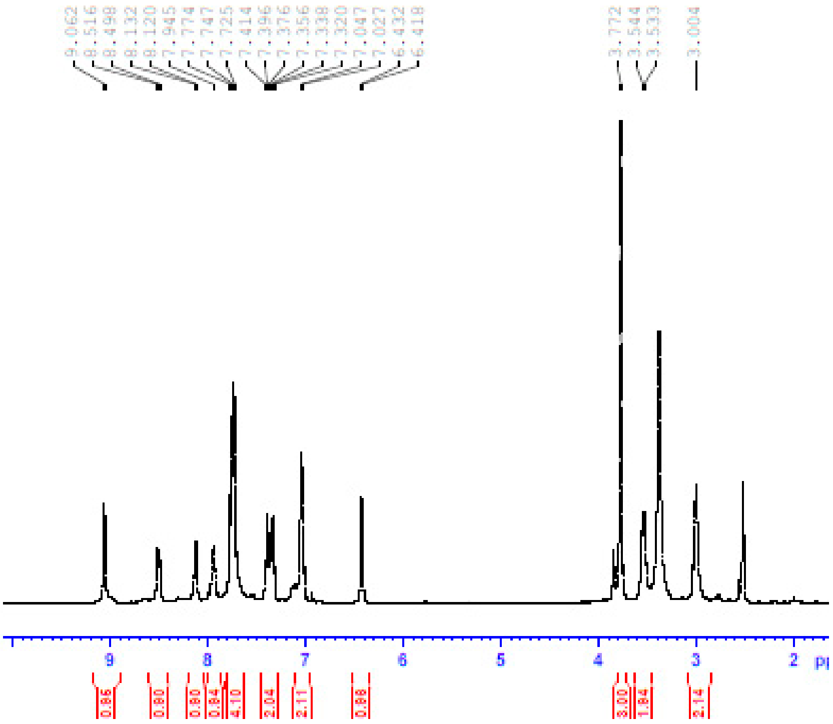
9.062
8.516
8.498
8.132
8.120
7.945
7.774
7.747
7.725
7.414
7.396
7.376
7.356
7.338
7.320
7.047
7.027
6.432
6.418
3.772
3.544
3.533
3.004
9
8
7
6
5
4
3
2
0.86
0.80
0.80
0.84
4.10
2.04
2.11
0.88
3.00
1.84
2.14

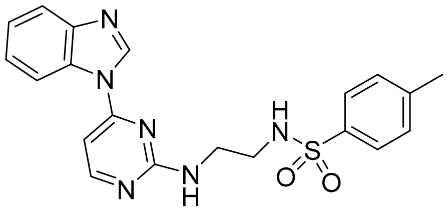

12g

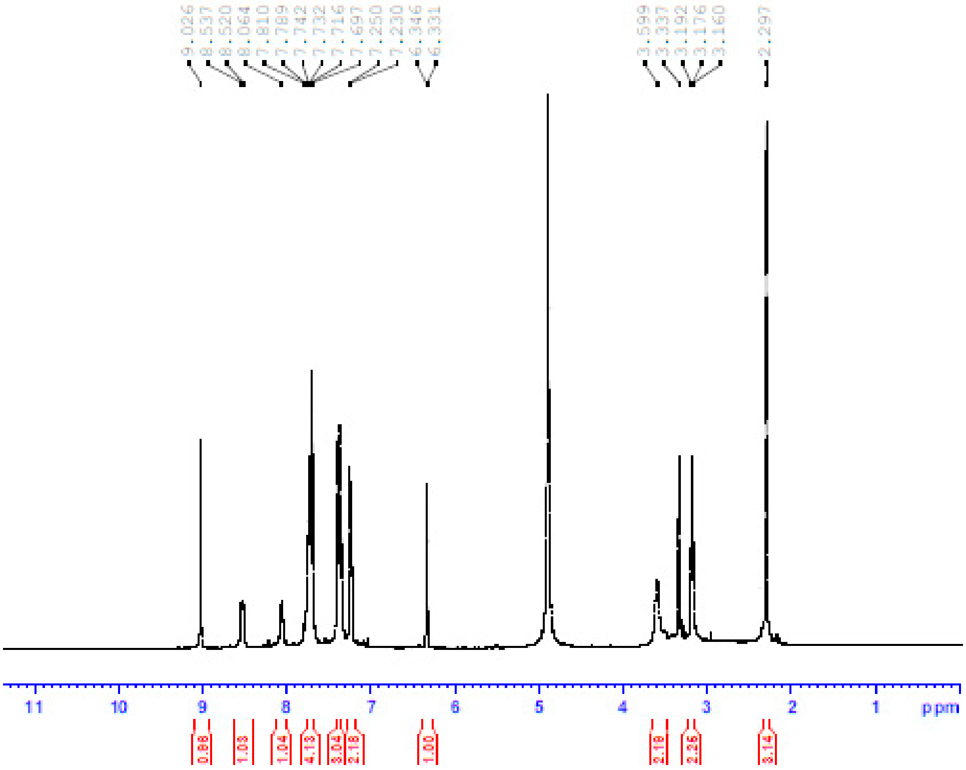
9.026
8.537
8.520
8.064
7.810
7.789
7.742
7.732
7.716
7.697
7.250
7.230
6.346
6.331
3.599
3.337
3.192
3.176
3.160
2.297
11 10 9 8 7 6 5 4 3 2 1 ppm
0.88
1.03
1.04
4.13
3.04
2.18
1.00
2.18
2.26
3.14

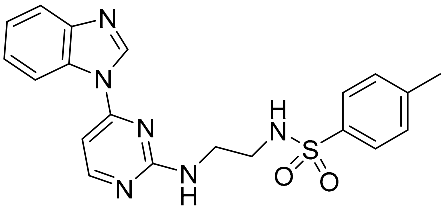

12g

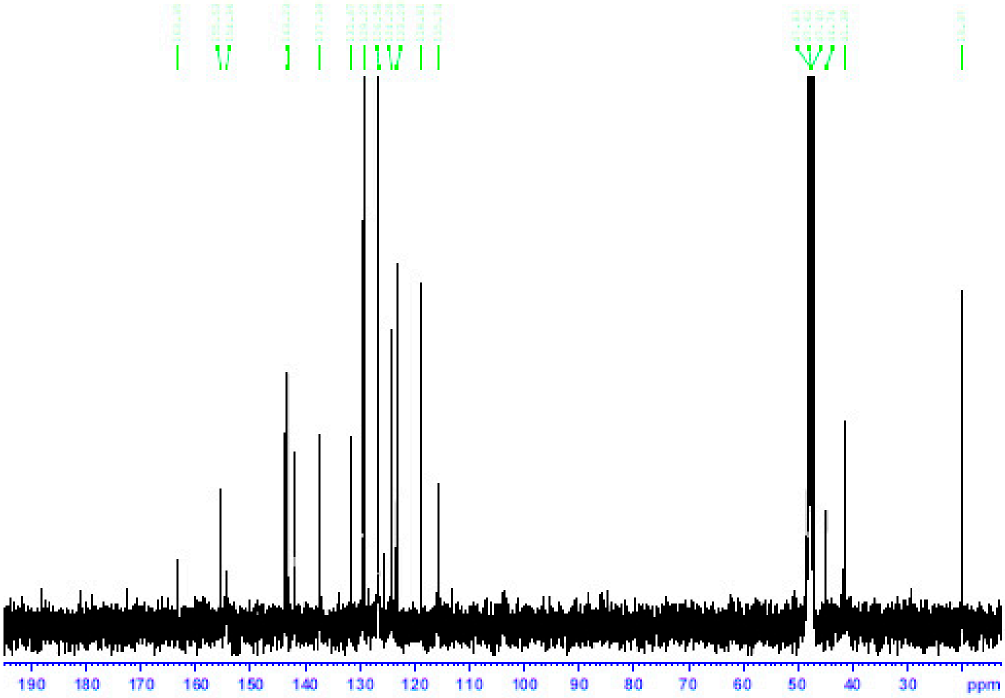

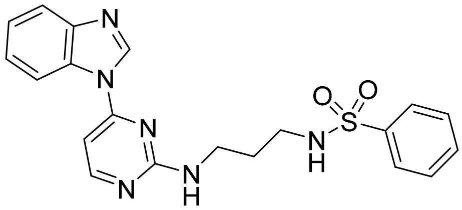

12h

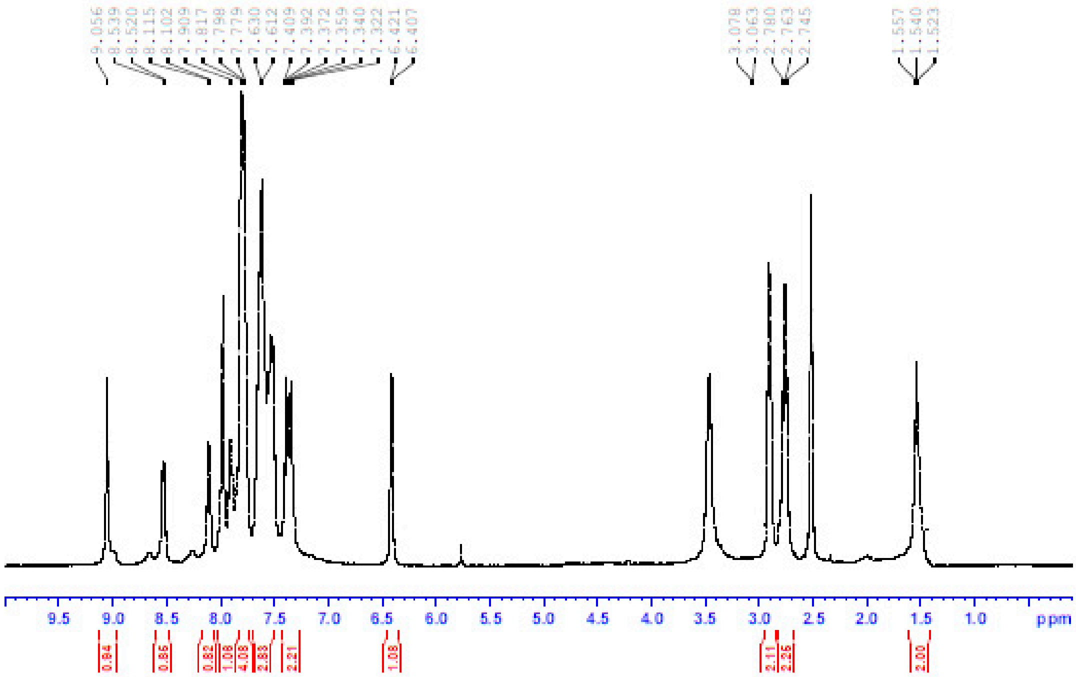

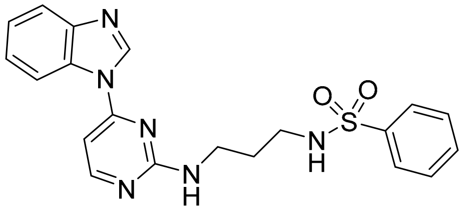

12h

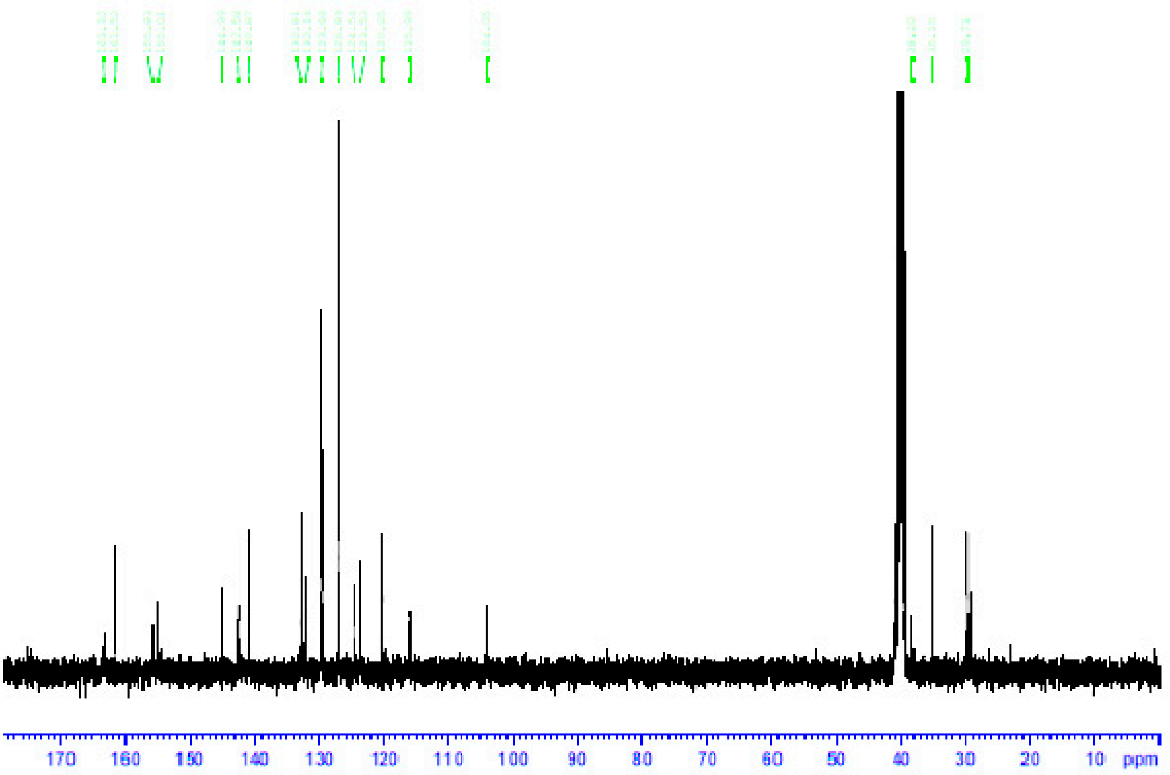

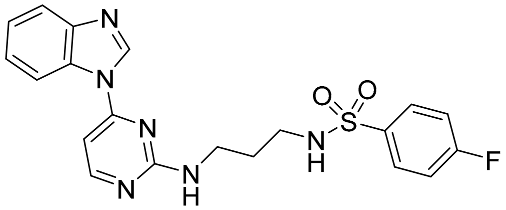

N
N
N
N
N
H
O
O
S
N
H
F

12i

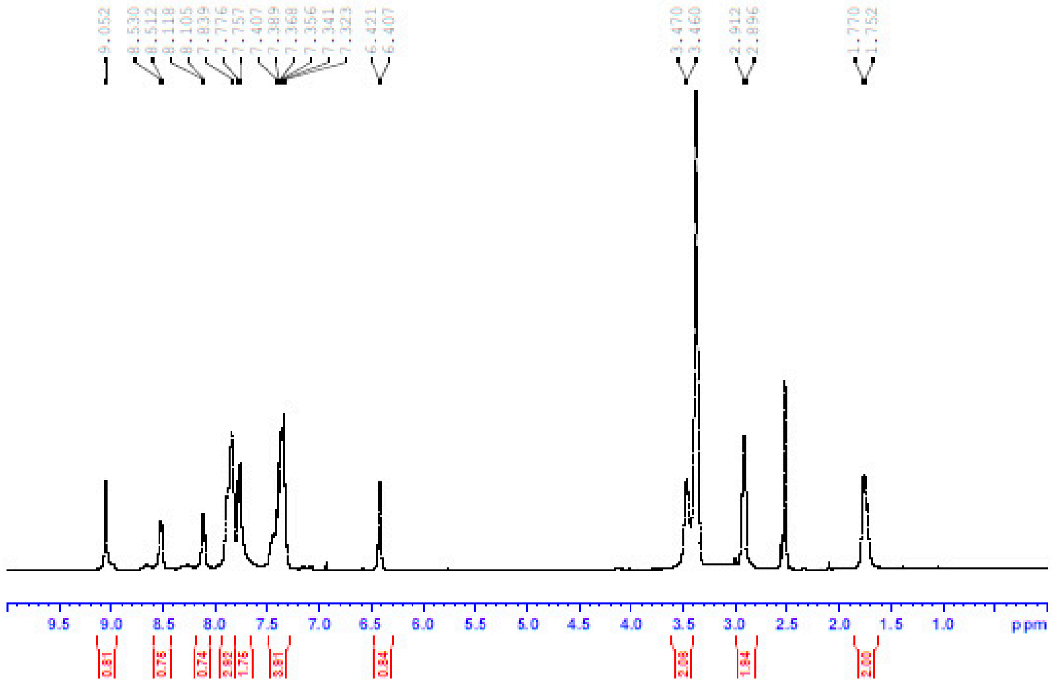

9.052
8.530
8.512
8.118
8.105
7.839
7.776
7.757
7.407
7.389
7.368
7.356
7.341
7.323
6.421
6.407
3.470
3.460
2.912
2.896
1.770
1.752
9.5 9.0 8.5 8.0 7.5 7.0 6.5 6.0 5.5 5.0 4.5 4.0 3.5 3.0 2.5 2.0 1.5 1.0 ppm

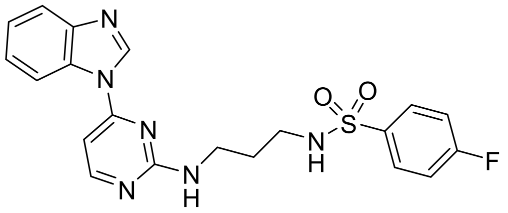
N
N
N
N
N
H
O
O
S
N
H
F

12i

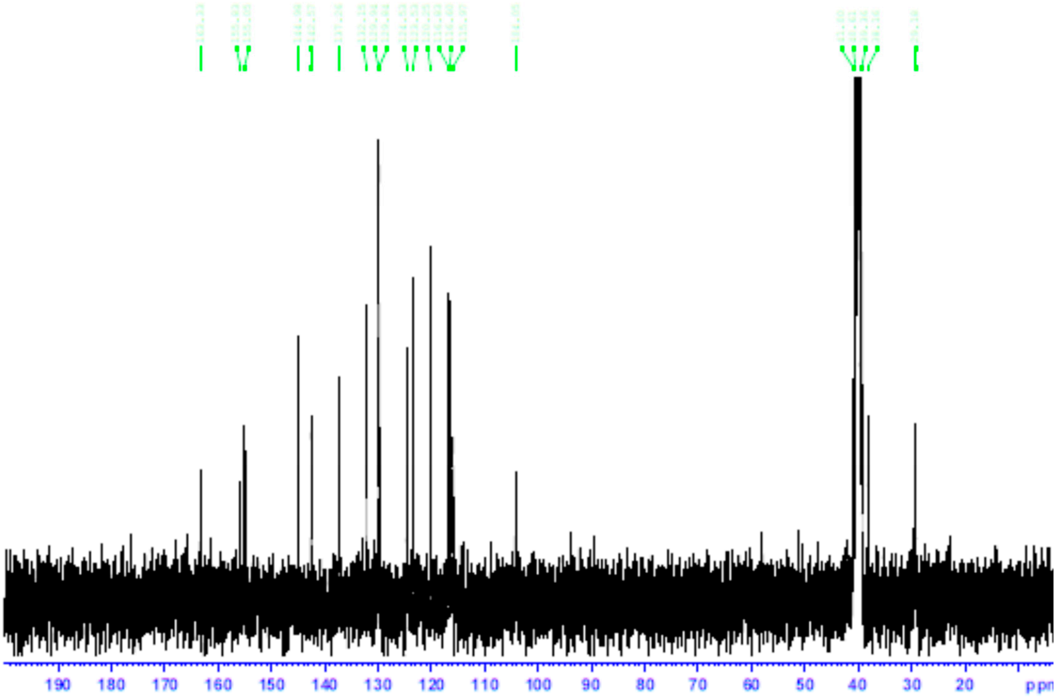
190 180 170 160 150 140 130 120 110 100 90 80 70 60 50 40 30 20 ppm

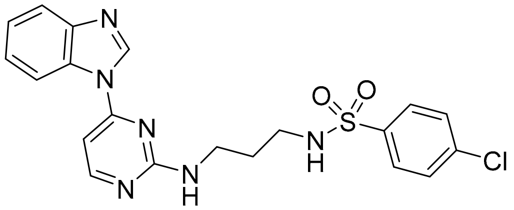

12j

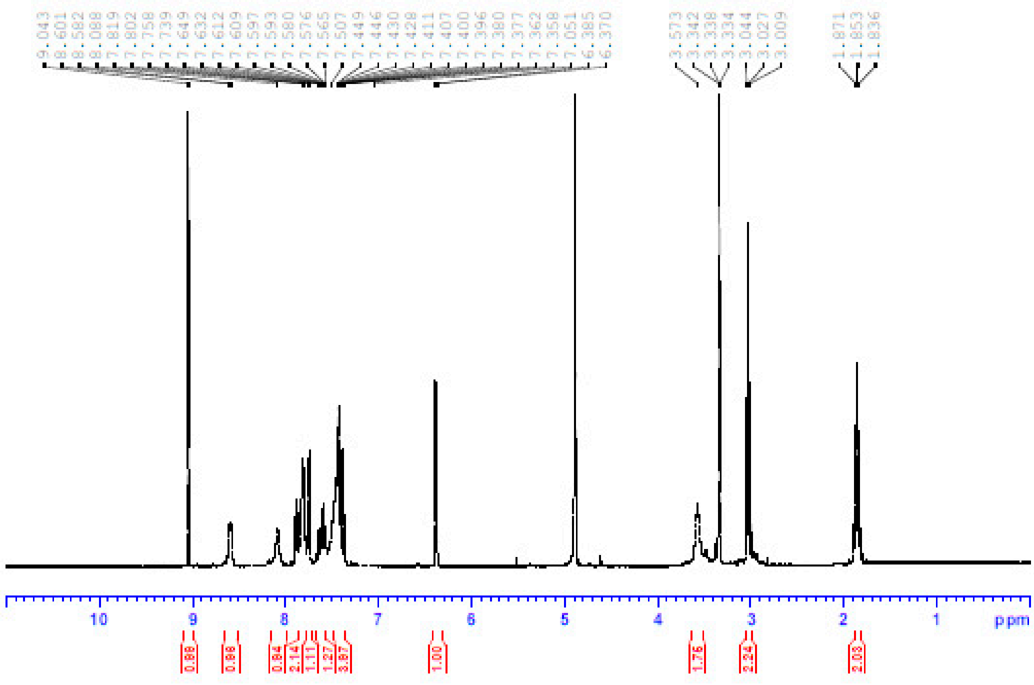

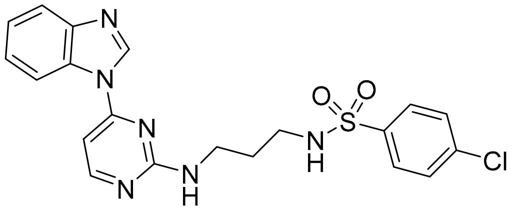

12j

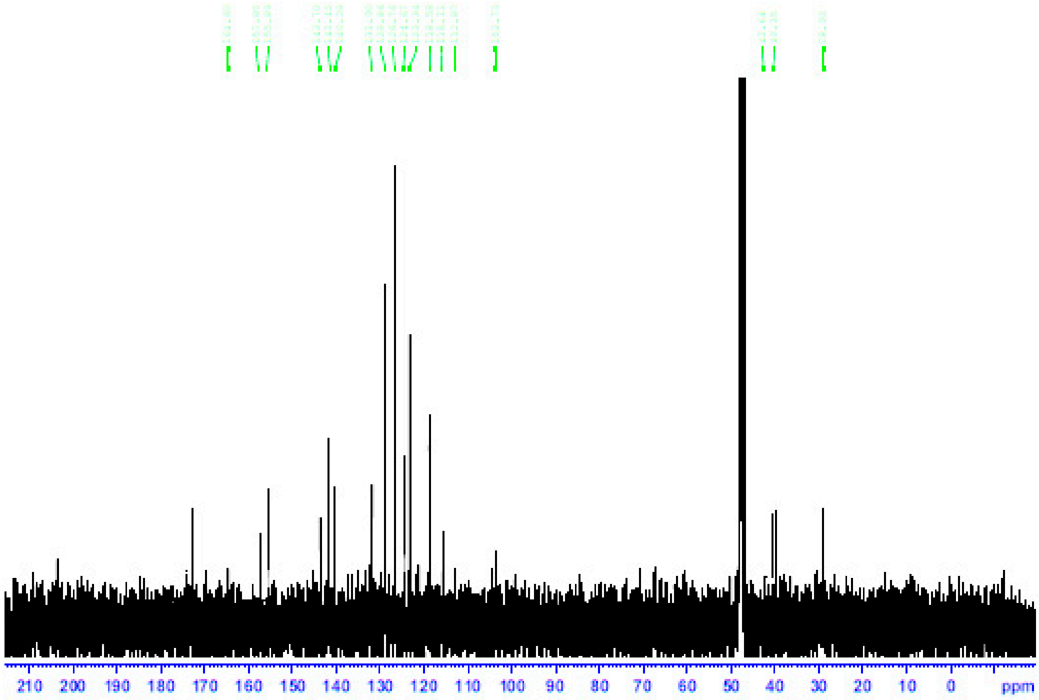
210 200 190 180 170 160 150 140 130 120 110 100 90 80 70 60 50 40 30 20 10 0 ppm

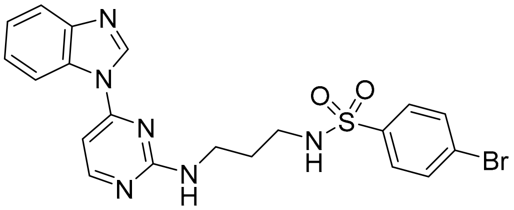

12k

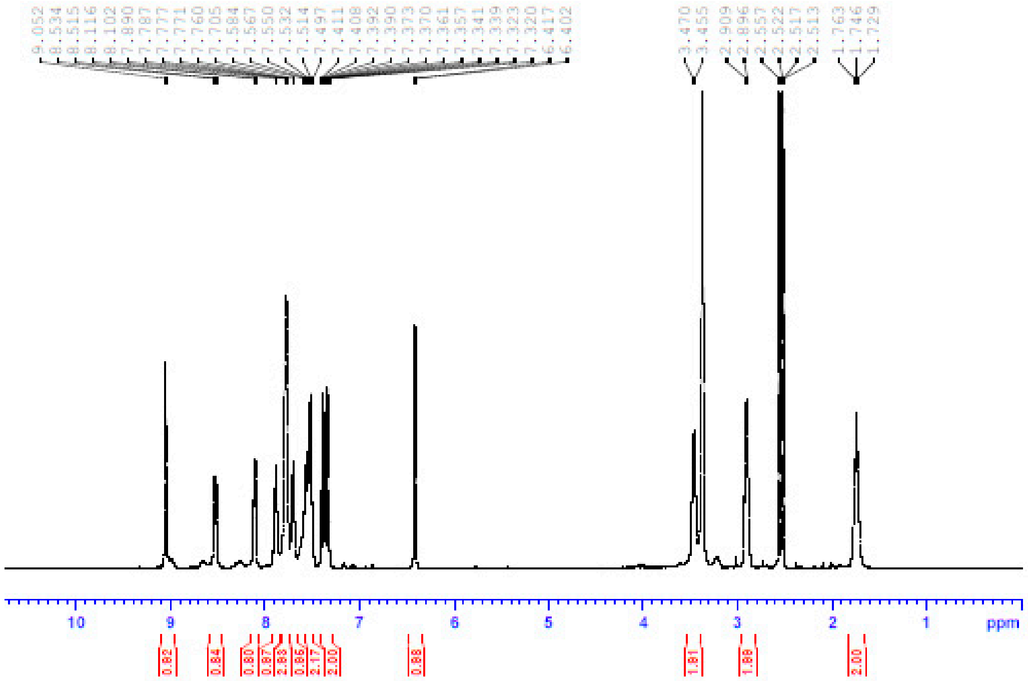

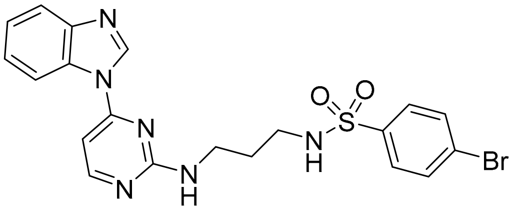

12k

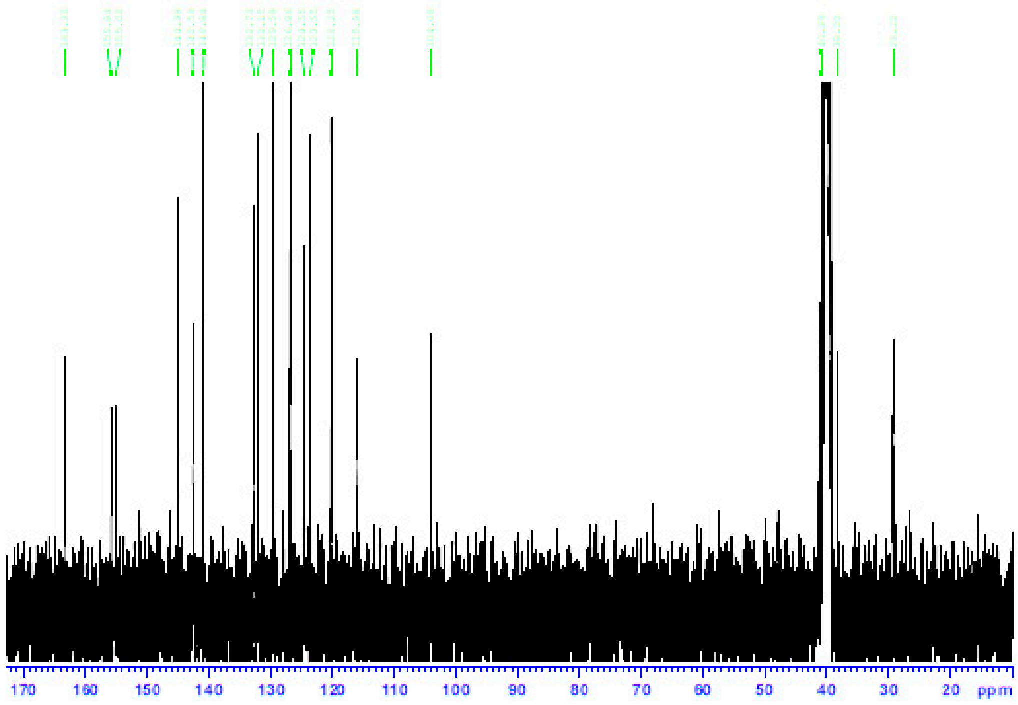

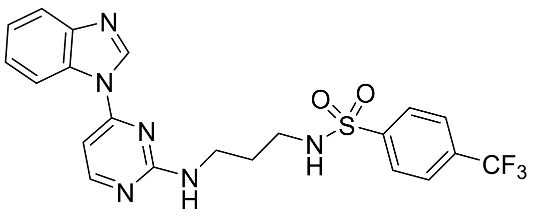

12l

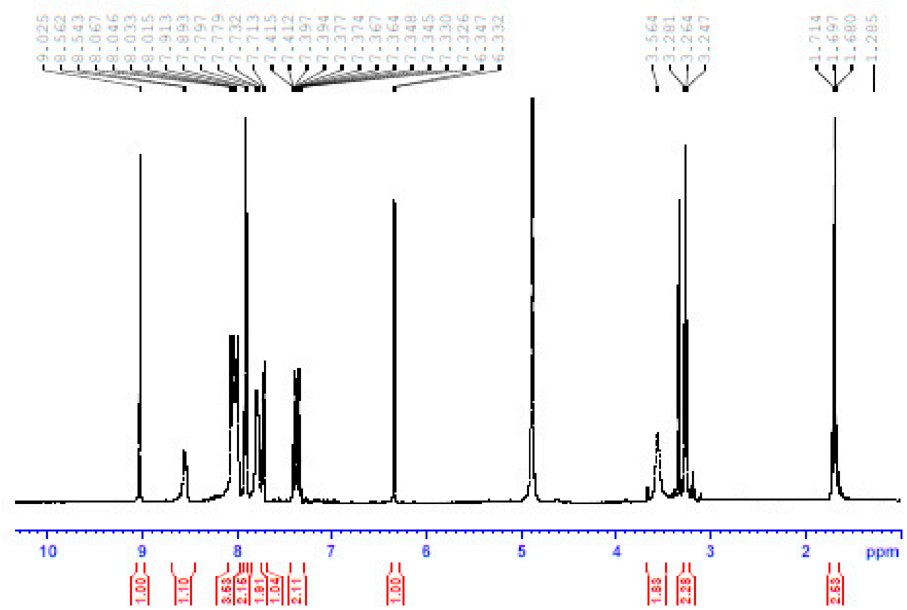

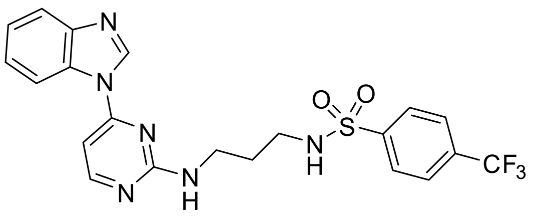

12l

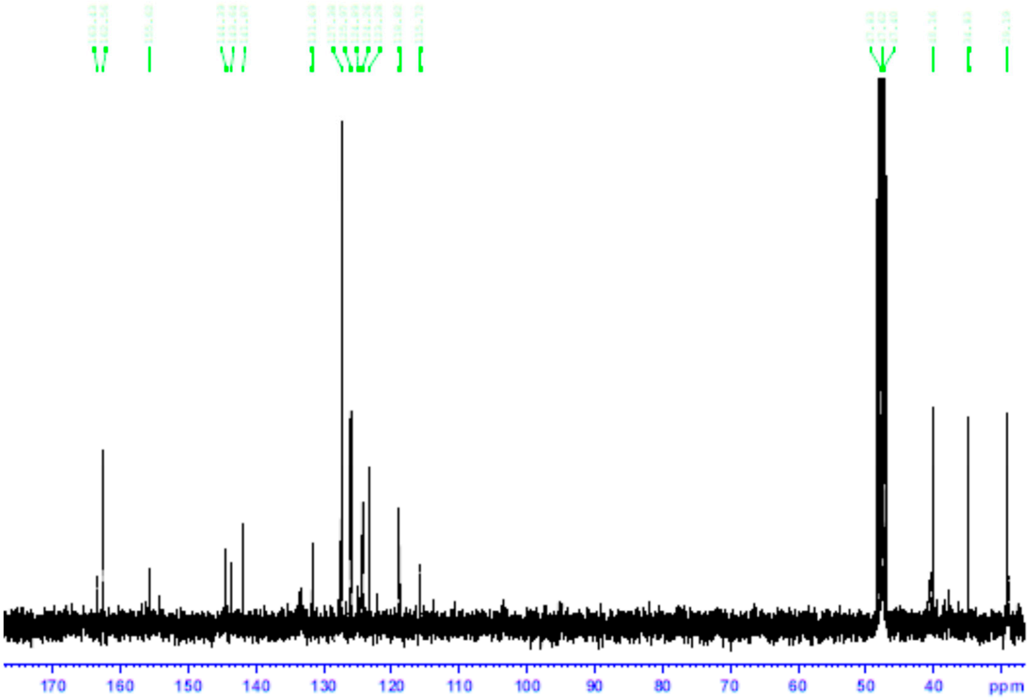

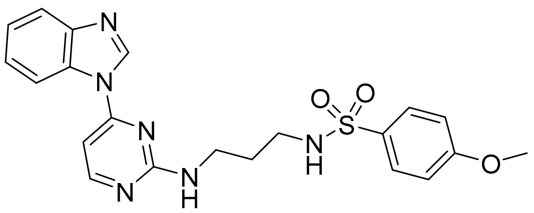

12m

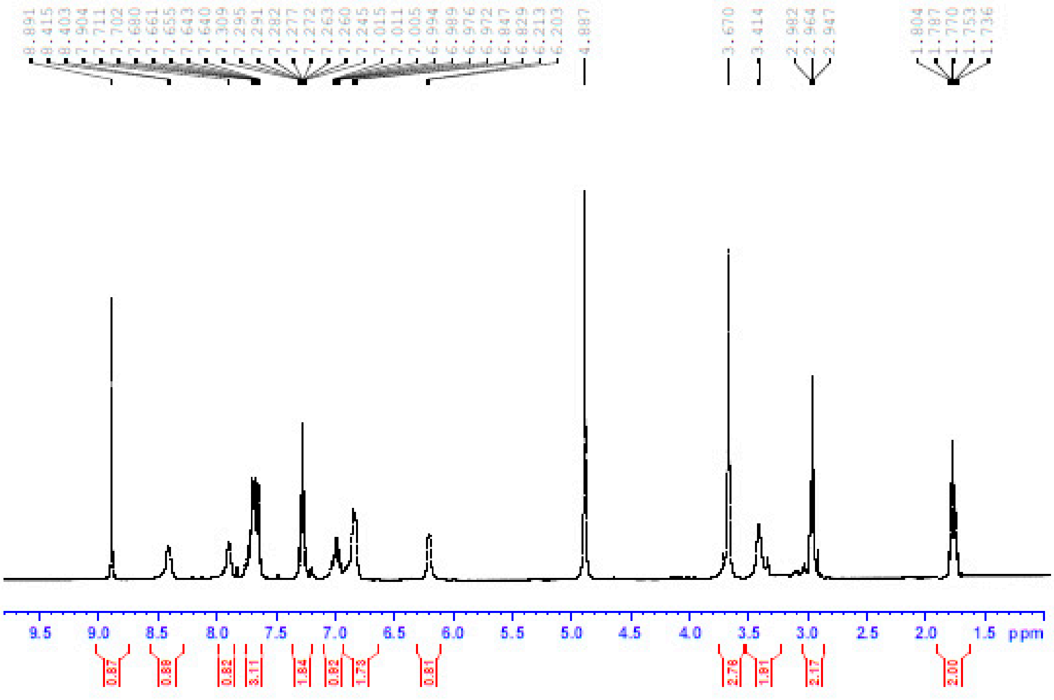

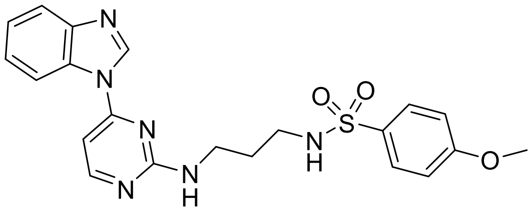

N
N
N
N
N
H
O
O
S
N
H
O

12m

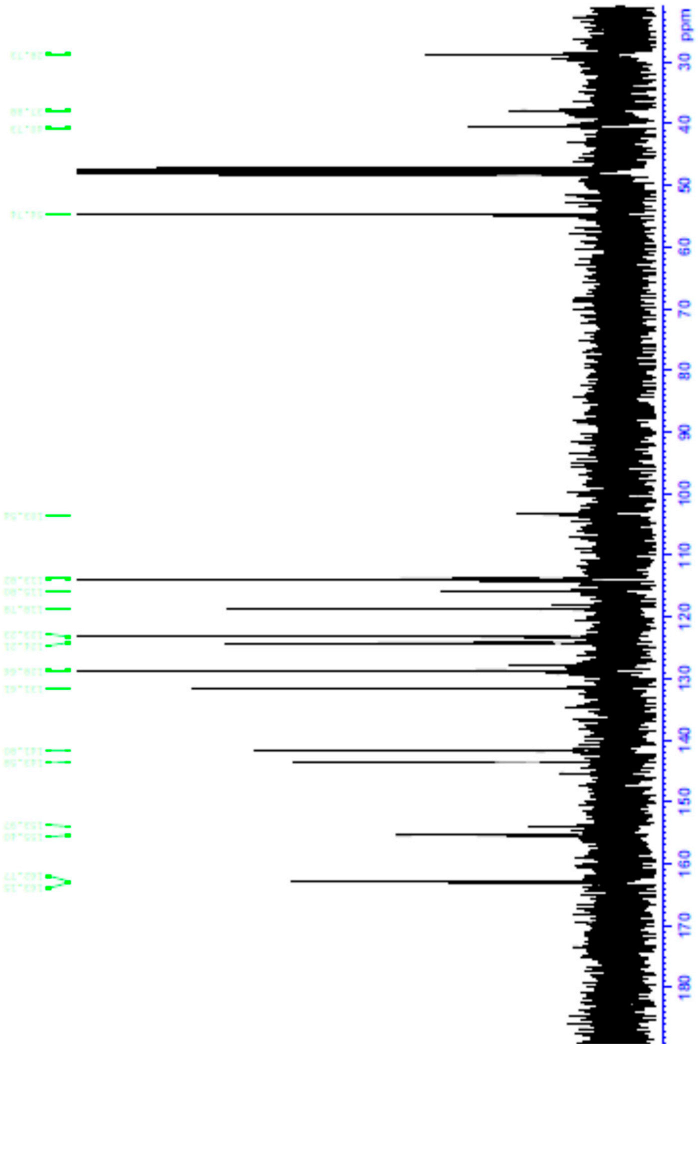

ppm
30
40
50
60
70
80
90
100
110
120
130
140
150
160
170
180

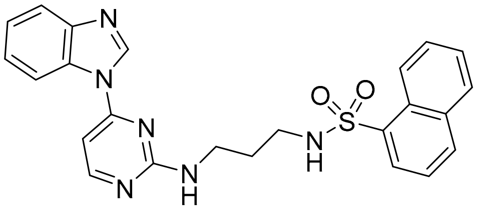

12n

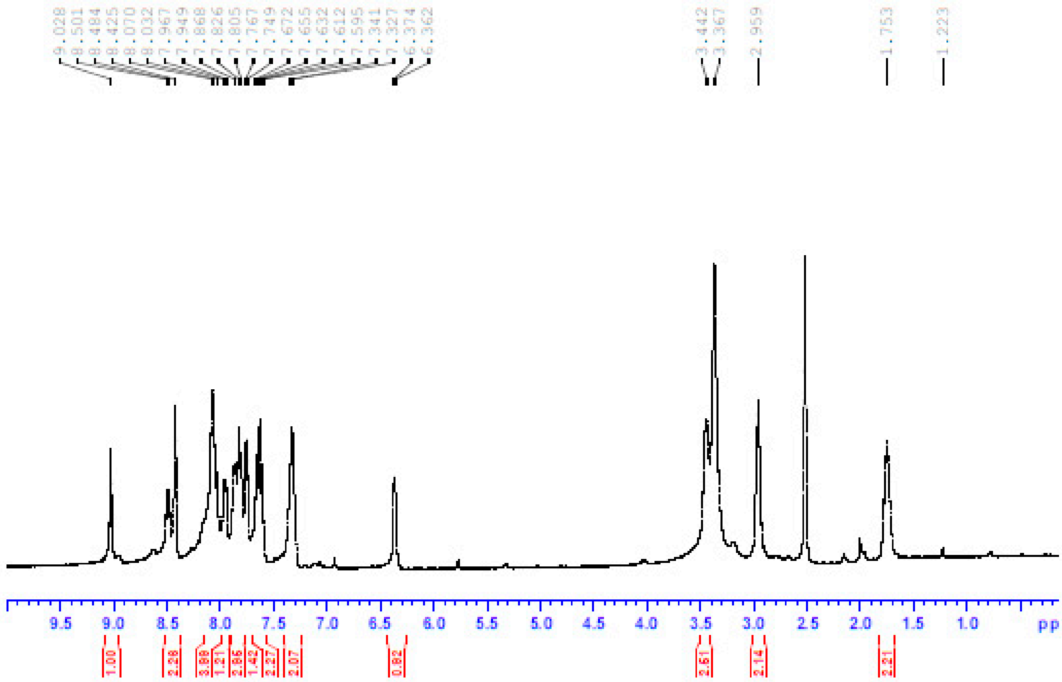

NCI standard procedure for 60 cell lines assay

The human tumor cell lines of the cancer screening panel are grown in RPMI 1640 medium containing 5% fetal bovine serum and 2 mM L-glutamine. For a typical screening experiment, cells are inoculated into 96 well microtiter plates in 100 µL at plating densities ranging from 5,000 to 40,000 cells/well depending on the doubling time of individual cell lines. After cell inoculation, the microtiter plates are incubated at 37° C, 5 % CO_2, 95 % air and 100 % relative humidity for 24 h prior to addition of experimental drugs.

After 24 h, two plates of each cell line are fixed in situ with TCA, to represent a measurement of the cell population for each cell line at the time of drug addition (Tz). Experimental drugs are solubilized in dimethyl sulfoxide at 400-fold the desired final maximum test concentration (10 µM) and stored frozen prior to use. At the time of drug addition, an aliquot of frozen concentrate is thawed and diluted to twice the desired final maximum test concentration with complete medium containing 50 µg/ml gentamicin. Additional four, 10-fold or ½ log serial dilutions are made to provide a total of five drug concentrations plus control. Aliquots of 100 µl of these different drug dilutions are added to the appropriate microtiter wells already containing 100 µl of medium, resulting in the required final drug concentrations.

Following drug addition, the plates are incubated for an additional 48 h at 37°C, 5 % CO_2, 95 % air, and 100 % relative humidity. For adherent cells, the assay is terminated by the addition of cold TCA. Cells are fixed in situ by the gentle addition of 50 µl of cold 50 % (w/v) TCA (final concentration, 10 % TCA) and incubated for 60 minutes at 4°C. The supernatant is discarded, and the plates are washed five times with tap water and air dried. Sulforhodamine B (SRB) solution (100 µl) at 0.4 % (w/v) in 1 % acetic acid is added to each well, and plates are incubated for 10 minutes at room temperature. After staining, unbound dye is removed by washing five times with 1 % acetic acid and the plates are air dried. Bound stain is subsequently solubilized with 10 mM trizma base, and the absorbance is read on an automated plate reader at a wavelength of 515 nm. For suspension cells, the methodology is the same except that the assay is terminated by fixing settled cells at the bottom of the wells by gently adding 50 µl of 80 % TCA (final concentration, 16 % TCA). Using the seven absorbance measurements [time zero, (Tz), control growth, (C), and test growth in the presence of drug at the five concentration levels (Ti)], the percentage growth is calculated at each of the drug concentrations levels. Percentage growth inhibition is calculated as:

[(Ti-Tz)/(C-Tz)] x 100 for concentrations for which Ti>/=Tz

[(Ti-Tz)/Tz] x 100 for concentrations for which Ti<Tz.

Three dose response parameters are calculated for each experimental agent. Growth inhibition of 50 % (GI50) is calculated from [(Ti-Tz)/(C-Tz)] x 100 = 50, which is the drug concentration resulting in a 50% reduction in the net protein increase (as measured by SRB staining) in control cells during the drug incubation. The drug concentration resulting in total growth inhibition (TGI) is calculated from Ti = Tz. The LC50 (concentration of drug resulting in a 50% reduction

in the measured protein at the end of the drug treatment as compared to that at the beginning) indicating a net loss of cells following treatment is calculated from [(Ti-Tz)/Tz] x 100 = -50. Values are calculated for each of these three parameters if the level of activity is reached; however, if the effect is not reached or is exceeded, the value for that parameter is expressed as greater or less than the maximum or minimum concentration tested.

Evaluation of the Antiproliferative Activity Against A375 and SK-MEL-5

A375 and SK-MEL-5 cells were purchased from American Type Culture Collection (ATCC, Rockville, MD, USA) and maintained in Dulbecco's modified eagle medium (DMEM, Welgene, Daegu, Republic of Korea) supplemented with 10% fetal bovine serum (FBS, Welgene, Daegu, Republic of Korea) and 1% penicillin/streptomycin (Welgene, Daegu, Republic of Korea) in a humidified atmosphere with 5% CO_2 at 37 °C. Both cells were taken from culture substrate with 0.05% trypsin-0.02% EDTA and plated at a density of 5 × 103 cells/well in 96 well plates and then incubated at 37 °C for 24 h in a humidified atmosphere with 5% CO_2 prior to treatment with various concentrations (3-fold serial dilution, 12 points) of the tested compounds. The cells were incubated for 48 h after treatment with the test compounds. The cell viability was assessed by the conventional 3-(4,5-dimethylthiazol-2-yl)-2,5-diphenyltetrazolium bromide (MTT) reduction assay. MTT assays were carried out with CellTiter 96® (Promega) according to the manufacturer's instructions. The absorbance at 590 nm was recorded using EnVision 2103 (Perkin Elmer; Boston, MA, USA). The IC_{50} values were calculated using GraphPad Prism 4.0 software.

Enzyme assay

Reaction Biology Corp. Kinase HotSpotTM service

http://www.reactionbiology.com was used for screening of compounds IC_{50} Profiler Express for IC_{50} measurement. Assay protocol: In a final reaction volume of 25 μL, kinase (5–10 mU) is incubated with 25 mM Tris pH 7.5, 0.02 mM EGTA, 0.66 mg/mL myelin basic protein, 10 mM magnesium acetate and [γ33P-ATP] (specific activity approx. 500 cpm/pmol, concentration as required). The reaction is initiated by the addition of the Mg-ATP mix. After incubation for 40 min at room temperature, the reaction is stopped by the addition of 5 μL of a 3% phosphoric acid solution. 10 μL of the reaction is then spotted onto a P30 filtermat, and washed three times for 5 min in 75 mM phosphoric acid and once in methanol prior to drying and scintillation counting.
